# T-cell and monocyte DNA methylation and Wnt dysregulation in common variable immunodeficiency: an exploratory study

**DOI:** 10.3389/fimmu.2026.1931297

**Published:** 2026-10-01

**Authors:** Vegard Myhre, Mingyi Yang, Mari Kaarbø, Mai Sasaki Aanensen Fraz, Annika E. Michelsen, Børre Fevang, Pål Aukrust, Thor Ueland, Magnar Bjørås, Silje Fjellgård Jørgensen

**Affiliations:** 1 Research Institute of Internal Medicine, Division of Surgery and Specialized Medicine, Oslo University Hospital, Oslo, Norway; 2 Institute of Clinical Medicine, University of Oslo, Oslo, Norway; 3 Department of Microbiology, Oslo University Hospital, Oslo, Norway; 4 Department of Medical Biochemistry, Oslo University Hospital, Oslo, Norway; 5 Section of Clinical Immunology and Infectious Diseases, Oslo University Hospital, Rikshospitalet, Oslo, Norway; 6 Faculty of Health Sciences, K.G. Jebsen Centre for Thrombosis Research (TREC), University of Tromsø, Tromsø, Norway; 7 Department of Clinical and Molecular Medicine, Norwegian University of Science and Technology, Norwegian University of Science and Technology, Trondheim, Norway; 8 Department of Microbiology, University of Oslo, Oslo, Norway

**Keywords:** autoimmunity, CpG methylation;, CVID - common variable immunodeficiency, epigenetic, inflammation, monocytes, T cells, Wnt signaling

## Abstract

**Background:**

Many patients with common variable immunodeficiency (CVID) develop inflammatory/autoimmune complications associated with T-cell and monocyte dysregulation which may involve epigenetic modifications.

**Objective:**

To characterize genome-wide CpG methylation in CD3^+^ T-cells and CD14^+^ monocytes in CVID and relate epigenetic alterations to gene expression and circulating proteins.

**Methods:**

Whole-genome bisulphite sequencing was performed on purified CD3^+^ T-cells and CD14^+^ monocytes from three CVID patients with inflammatory complications and three healthy controls per cell type. Differentially methylated cytosines were identified and analysed by pathway over-representation. Selected pathway-related proteins were measured in plasma from 60 CVID patients and 30 controls and evaluated in three public CVID transcriptomic datasets.

**Results:**

In this small discovery cohort, CVID was associated with widespread, cell type-specific methylation differences in T-cells and monocytes. T-cells showed a predominance of hypomethylated cytosines and regions, whereas monocytes displayed a more mixed pattern with roughly balanced hypo- and hypermethylation at individual sites. In T-cells, pathway analysis identified Wnt signalling, T-cell differentiation and actin organisation among the top differentially methylated processes. As Wnt signalling is underexplored in CVID and key Wnt modulators are measurable in plasma, we measured Wnt-related proteins in a larger CVID cohort and controls. In plasma, the Wnt antagonists DKK1 and DKK3 were reduced and Wnt agonist RSPO3 increased in CVID. Analyses of three public CVID transcriptomic datasets showed differential expression of Wnt-related genes. Monocyte pathway signals, mainly related to cell adhesion and actin organisation, were more heterogeneous and should be regarded as exploratory given the very small discovery cohort and the inclusion of a patient with a TNFAIP3 variant.

**Conclusion:**

In this explorative study we found that CVID is associated with extensive, cell type-specific epigenetic remodelling, particularly in T cells. Wnt signalling emerges as a recurrently altered pathway across DNA methylation, circulating proteins and gene expression, supporting Wnt-related mechanisms as a potential contributor to CVID immunopathology that should be further explored.

## Introduction

Common variable immunodeficiency (CVID) is the most common symptomatic primary immunodeficiency in adults, with an estimated prevalence of 1 in 25, 000-50, 000 (1, 2). CVID is characterized by impaired B-cell function leading to decreased immunoglobulin (Ig) production with reduced levels of IgG and IgA, leading to recurrent airway infections with capsulated bacteria (3, 4). Up to 70% of patients also have inflammatory and autoimmune complications, associated with increased morbidity and mortality. These complications appear to be related to dysregulated T-cell and monocyte/macrophage responses (2, 5, 6). Patients with CVID and autoimmune or inflammatory manifestations frequently display a skewed T-cell profile, characterized by enhanced activity of pro-inflammatory T helper cell type 1 cells (Th1), and a reduced number of regulatory T-cells (Treg) (7–9). Moreover, several studies suggest that monocytes/macrophages contribute to these complications through increased production of pro-inflammatory cytokines (10–12). Thus, although B-cell pathology is the hallmark of CVID, persistent activation and functional abnormalities of T-cells and monocytes/macrophages represent critical drivers of the inflammatory and autoimmune phenotype in CVID and may aggravate both immune dysregulation and organ pathology (11, 13).

We have recently suggested a role of epigenetic DNA methylation in the aetiology of duodenal inflammation of CVID patients (14). In addition, epigenetic modifications have been proposed as a potential mechanism underlying altered B-cell responses in CVID (15–18). In general, however, data on epigenetic changes in CVID remain limited. To the best of our knowledge, studies specifically addressing epigenetic modifications in relation to T-cell and monocyte pathology are lacking.

CpG methylation is a key epigenetic modification where cytosine is converted to 5-methylcytosine (5mC) in DNA. This conversion occurs at 5’-cytosine-phosphate-guanine-3’ (CpG) dinucleotides, and the resulting methylation patterns can be inherited through cell divisions, thereby marking specific cell lineages, but can also change dynamically in response to stimuli such as infections, inflammation, and environmental exposures (19, 20). CpG methylation can interfere with transcription factor binding and other regulatory interactions with DNA, and is therefore an important regulator of gene expression, cell differentiation and immune cell function, acting beyond the inherited DNA (21–23).

Given the proposed role of T-cells and monocytes in the pathogenesis of autoimmune and inflammatory complications in CVID, we examined the CpG methylation profile in CD3^+^ T-cells and CD14^+^ monocytes from this subgroup of CVID patients. Proteins related to the pathways that were most affected by epigenetic regulation were subsequently measured in plasma from a larger CVID cohort. In addition, we explored the relevance of these pathways at transcriptional level by analysing RNA expression data from three independent publicly available CVID cohorts.

## Methods

### Study design

In total, six CVID patients were included in the epigenetic analyses (three contributing CD3^+^ T-cells and another three contributing CD14^+^ monocytes, with no overlap between the patient groups), all enrolled from the Outpatient Clinic at Oslo University Hospital between 2003 and 2005. At inclusion, none had acute infection or exacerbation of a chronic infection, and none received prophylactic antibiotics or immunosuppressive therapy, apart from Ig replacement. CVID was diagnosed according to the 1999 WHO criteria (24), which were the prevailing diagnostic criteria at inclusion. In a retrospective review in 2025, all surviving patients fulfilled the 2019 ESID criteria (25). Age-matched healthy controls (n=6) based on medical history and absence of regular medication were recruited from the same period. All six CVID patients in the WGBS discovery cohort belonged to the non-infectious complication (“complicated CVID”) subgroup and there is no overlap between these patients (**Table 1**) and those in the plasma cohort (**Table 2**). Patients were retrospectively screened for monogenic variants using a targeted panel of 598 genes associated with primary immunodeficiency and haematological diseases (https://www.genetikkportalen.no/find-ngs/1100, version 4), which was applied years after recruitment of the discovery cohort.

**Table 1:** Characteristics of the CpG study cohort.

Characteristic	CVID	Controls	P-value
CD3			
Sample size, n	3	3	
Age (years), mean (min-max)	38 (24-46)	33 (24-43)	0.51^a^
Female, n	2	2	1.00^b^
CD14			
Sample size, n	3	3	
Age (years), mean (min-max)	35 (22-46)	30 (24-43)	0.83^a^
Female, n	1	2	1.00^b^

CVID, Common variable immunodeficiency; ^a^Mann Whitney; ^b^Fisher’s exact. No shared patients with the plasma Wnt protein cohort in **Table 2**.

**Table 2:** Characteristics of the Wnt study cohort.

Characteristic	CVID	Controls	P-value
Sample size, n	60	30	-
Ageinyears, mean (SD)	43 (14)	44 (15)	0.95^a^
Female, n (%)	37 (63)	18 (60)	1.00^b^
BMI, mean (SD)	25 (5)	25 (3)	0.80^a^
Infection only, n (%)	13 (22)	-	-
Complications, n (%)	47 (78)	-	-
*Splenomegaly*, *n (%)*	39 (83)	-	-
*Lymphadenopathy*, *n (%)*	36 (77)	-	-
*GLILD*, *n (%)*	23 (49)	-	-
*GI disease*, *n (%)*	22 (47)	-	-
*Granulomas*, *n (%)*	13 (28)	-	-
*Liver disease*, *n (%)*	11 (23)	-	-
*Other autoimmunity*, *n (%)*	11 (23)	-	-
*Autoimmune cytopenia*, *n (%)*	9 (19)	-	-

CVID, Common variable immunodeficiency; BMI, body mass index; GLILD, granulomatous interstitial lung disease; GI, gastrointestinal. Text in italics indicates subgroups of complications. A patient may have one or more complications.

^a^ Mann Whitney; ^b^Fisher’s exact. No shared patients with the WGBS discovery cohort in **Table 1**.

The plasma cohort was recruited at the same department between 2021 and 2024 and included 60 CVID patients fulfilling the 2019 ESID criteria (25) and 30 age-matched healthy controls. As in the epigenetic study, individuals with acute infection, exacerbation of inflammatory/autoimmune disease, or ongoing immunomodulatory therapy (other than Ig replacement) at enrolment were excluded. In the plasma cohort, criteria: patients were classified as “non-infectious complications” (i.e., presence of one or more of the following: enteropathy, splenomegaly, lymphoid hyperplasia, organ-specific autoimmunity, autoimmune cytopenia, granulomas, granulomatous lymphocytic interstitial lung disease, nodular regenerative hyperplasia, or lymphoma), or as “infection only” (i.e., only recurrent bacterial infections in the respiratory tract and absence of the above-mentioned complications), based on previously defined criteria (2), with two exceptions; CVID enteropathy was defined as biopsy-proven lymphocytic infiltrations and/or persistent diarrhoea after exclusion of GI infection and CT-defined granulomatous interstitial lung disease (GLILD) instead of the previous term lymphoid interstitial pneumonitis (26).

### Cell isolation

Peripheral blood mononuclear cells (PBMC) were isolated from sodium-heparin anticoagulated blood by density-gradient centrifugation using Lymphoprep (Axis-Shield, Oslo, Norway) within one hour of sampling. CD14^+^ monocytes were obtained by positive selection with anti-CD14 Dynabeads, and CD3^+^ T-cells by negative selection (T-cell negative isolation kit; both from Dynal, Oslo, Norway). Briefly, PBMCs suspended in PBS with 0.3% BSA were mixed with monodisperse immunomagnetic beads against CD14 (for positive selection of CD14^+^ monocytes) and CD14/CD19/CD56 (for negative selection of CD3^+^ T-cells) in a cell-to-bead ratio of 1:5. Rosetting cells were isolated magnetically after 30 minutes for either storage (positive selection of monocytes) or disposal (negative selection of T-cells) and the non rosetting fraction (enriched for CD3^+^ T cells) was collected. Purity was verified by flow cytometry (27–29). The monocyte fraction contained >95% CD14^+^ cells, and the T-cell fraction >90% CD3^+^ cells (27). The isolated cells were immediately cryopreserved in liquid nitrogen and remained continuously frozen without repeated freeze-thaw cycles until DNA extraction for WGBS. CVID and control samples were collected, processed and stored identically. DNA quantity and integrity were systematically assessed after thawing, with all WGBS samples showing excellent DNA integrity (DIN 9.0-9.8) (30), indicating minimal degradation despite long-term storage.

### Whole-genome bisulphite sequencing

DNA was isolated from frozen cell pellets using the Allprep DNA/RNA/Protein kit (Qiagen, Hilden, Germany) according to the manufacturer’s instructions with minor modifications. WGBS was performed by Admera Health (NJ, USA), including bisulphite conversion (EZ DNA Methylation-Gold Kit), Accel-NGS Methyl-Seq library preparation and Illumina HiSeq sequencing (150 bp paired-end, approximately 300 million read pairs per sample). Raw reads were quality-filtered and aligned to the hg38 reference genome, and genome-wide 5mC counts were extracted using standard pipelines; full laboratory and bioinformatic details are provided in **Supplementary Information**.

### Plasma analysis of Wnt-related proteins

Blood was collected in chilled EDTA tubes, centrifuged within 15 minutes at 2000g for 20 minutes to obtain platelet-poor plasma, aliquoted and stored at -80 °C until a single freeze-thaw. Plasma levels of Dickkopf-1 (DKK1), DKK3, secreted frizzled-related protein 3 (sFRP3), Wnt inhibitory factor (WIF) and R-spondin-3 (RSPO3) were measured by enzyme immunoassays (R&D Systems, Minneapolis, MN, USA). Assays were run in 384-well format using a SELMA pipetting robot and a BioTek dispenser/washer, and absorption was read at 450 nm with 540 nm reference. Intra- and inter-assay coefficients of variation were <10% for all measurements.

### Wnt expression in previous CVID studies

Publicly available mRNA expression data from three studies on CVID and T-cells or PBMC (15, 31, 32) were downloaded and analysed (**Supplementary Information**). Analyses were restricted to 355 genes annotated to the Gene Ontology “Wnt signalling pathway” (GO:0016055) (33).

### Statistical analyses

Bioinformatic processing of WGBS and transcriptomic data is outlined in **Supplementary Figure S1** and **Supplementary Information**. Differentially methylated CpG cytosines (DMCs), regions (DMRs) and promoters (DMPs) were identified using R (v4.4.3) with RStudio (v2023.06.0 + 421) and the methylKit (v1.34.0) package, and using Benjamini-Hochberg adjusted p-value <0.05 and a minimum absolute methylation difference of 10%. High- frequency DMCs was defined as subset of DMCs with additional thresholds; at least two DMCs at TSS +- 10kb, could be annotated to a gene, and with a mean methylation difference of at least |25%|. Over-representation analyses (ORA) were performed with Gene Ontology and BH corrected p-values. For each cell type, all significant DMCs, DMRs and DMPs were annotated to genes, and the union of these gene annotations was used as the input gene list for ORA. The background gene set consisted of all genes with at least one CpG site, region or promoter that passed quality filters and was tested for differential methylation in the WGBS data (see **Supplementary Information** for additional analytical details).

Group differences in plasma Wnt-related proteins were assessed in the total cohort (CVID_all vs. healthy controls) and in subgroup analyses (CVID_IO vs. healthy controls, CVID_NIC vs. healthy controls). For each analyte and comparison, distributions were evaluated using the Shapiro-Wilk test. If values were approximately normally distributed (i.e. Shapiro-Wilk > 0.05), an unpaired two-sample t test was applied (this was the case only for DKK1). For skewed analytes, data were log10-transformed and retested for normality; if the log-transformed values were approximately normal, group differences were analysed using t tests on the transformed data, whereas persistently non-normal distributions were compared using Mann-Whitney U tests. Analytes with skewed distributions are plotted on a log10 scale, whereas approximately normally distributed analytes are shown on a linear scale.

## Results

### Clinical characteristics

The epigenetic (CpG) analyses were performed on CD3^+^ T-cells and CD14^+^ monocytes from three CVID patients with autoimmune/inflammatory complications and three healthy controls in each cell population, corresponding to six different patients and six matched controls in total (**Table 1**). In the CD14^+^ cohort, one CVID patient was later found to carry a monogenic TNFAIP3 variant, while the CD3^+^ T-cell cohort included three CVID patients without identified monogenic variants. This TNFAIP3 variant was first described in a CVID patient in 2020 (34) and was therefore not included in the original gene panels. No other CVID patients in this cohort had pathogenic variants detected by the gene panel.

### CpG methylation patterns separate CVID from healthy controls

CpG site coverages showed the highest frequency at about five reads per CpG site, with a genome-wide CpG coverage range from 12 to 35 million sites per sample, after read filtering (**Supplementary Figure S2, S3, S4A**). More than 1.6 million CpG sites were included in the analysis for both cell types, in addition to over 750, 000 CpG regions and over 72, 000 promoters (**Supplementary Figure S4B**). These data were then used to compare CpG methylation patterns between CVID patients and healthy controls.

We first performed hierarchical clustering using all retained CpG regions in CD3^+^ T cells and CD14^+^ monocytes, which provided a global overview but showed limited visual separation between CVID and healthy controls (**Supplementary Figure S5A-B**). In contrast, hierarchical clustering based on all identified DMRs in each cell type yielded a clear separation of the groups consistent with the use of regions showing statistically significant methylation differences (**Supplementary Figure S5C-D**). Partial least squares discriminant analysis (PLS-DA), applied as a supervised approach to maximize separation between groups, also showed a similar separation between CVID patients and healthy controls in both cell types (**Figures 1A, B**).

**Figure 1:**
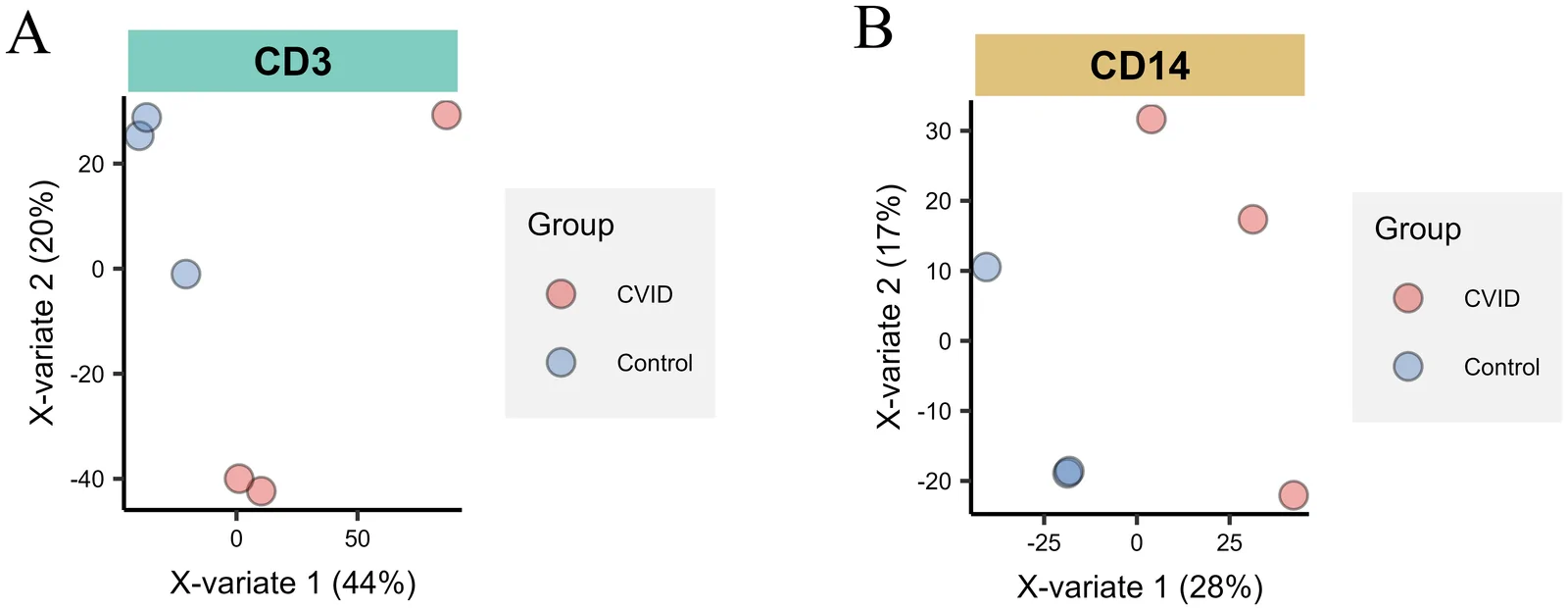
Partial least squares discriminant analysis (PLS-DA) of CpG methylation in CD3^+^ T-cells and CD14^+^ monocytes from CVID patients with non-infectious complications versus healthy controls. **(A)** PLS-DA of CD3^+^ T-cells between CVID and healthy controls. 805, 722 methylation regions were included in the analysis **(B)** PLS-DA of CD14^+^ monocytes; 776, 336 methylation regions were included in the analysis. In panel B, two control samples (blue circles, bottom left) heavily overlap and appear on top of each other.

### Differentially methylated CpG sites, regions and promoters in CD3^+^ T-cells in CVID

To pinpoint where these epigenetic differences occur in the genome, differential CpG methylation was next assessed at three levels: (i) individual CpG cytosines showing significant methylation differences between groups (DMCs), (ii) genomic regions containing clustered CpGs that were consistently hyper- or hypomethylated (DMRs), and (iii) promoter-associated CpG sites identified as differentially methylated (DMPs).

In T-cells, comparison of CVID patients to healthy controls revealed 486 DMCs and 3, 723 DMRs (**Figure 2A**). Approximately two thirds of these DMCs and DMRs were hypomethylated in CVID, which may reflect enhanced activation, whereas the remaining one-third were hypermethylated, which may reflect suppressed activation (35). For promoter-associated probes, 420 DMPs were detected in CVID CD3^+^ cells compared to controls. In contrast to DMCs/DMRs, roughly two-thirds of the DMPs were hypermethylated and one-third hypomethylated (**Figure 2A**). This contrasting pattern between DMCs/DMRs and DMPs was statistically supported by chi-square tests comparing the proportions of hyper- and hypomethylated sites across these categories (**Supplementary Figure S6**). The majority of DMCs and DMRs localized to intergenic or intronic DNA regions, whereas promoter and exon location were underrepresented (**Figure 2B**). Because DMPs are by definition promoter-associated, this difference in genomic distribution may partly account for the divergent hyper-to hypomethylation balance observed between DMCs/DMRs and DMPs.

**Figure 2:**
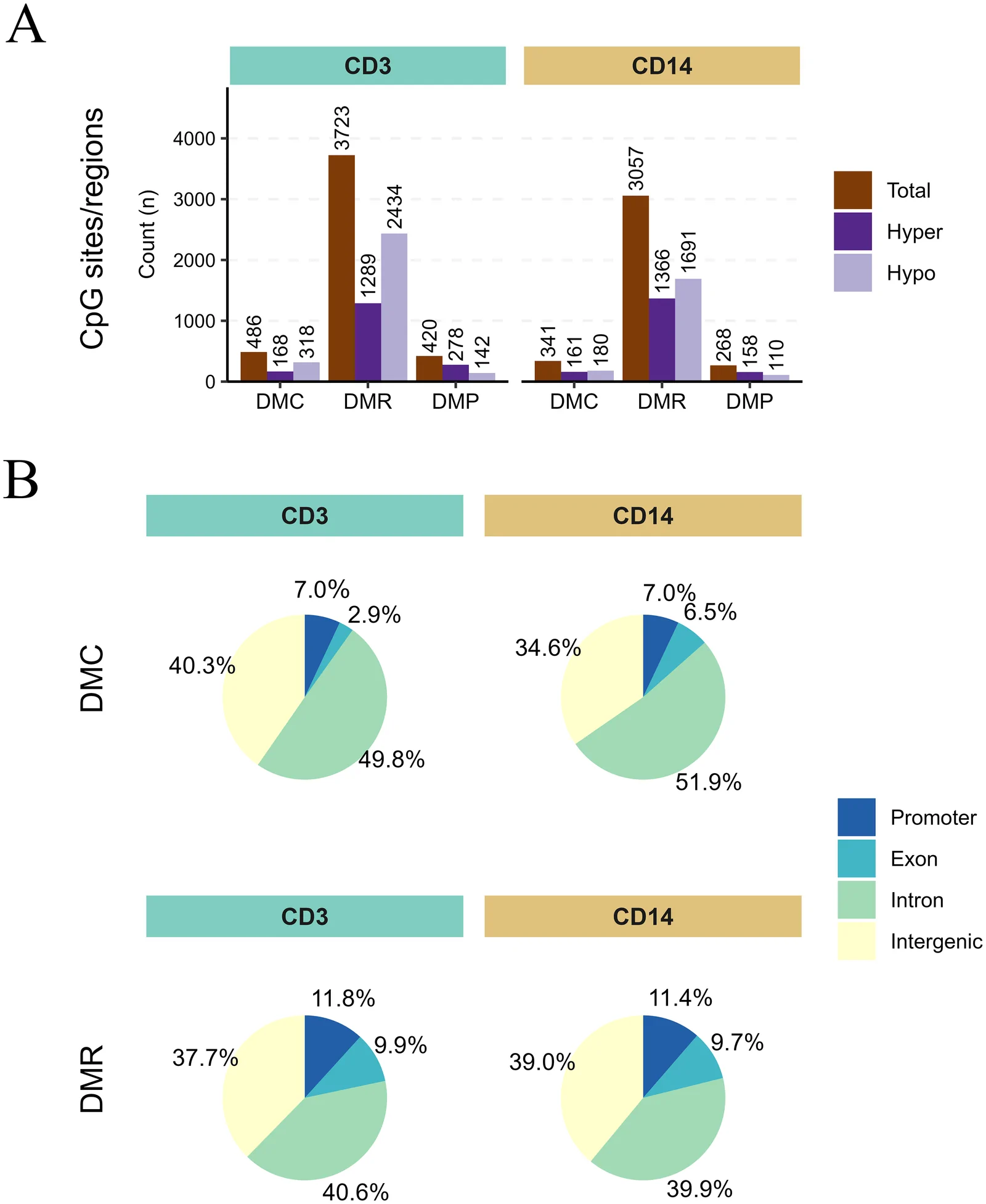
Distribution of significant differential methylation in CD3^+^ T-cells and CD14^+^ monocytes between CVID and healthy controls. **(A)** Total number of significant DMCs, DMRs, and DMPs by cell type, subdivided by hypermethylated and hypomethylated events **(B)** Genomic distribution of DMCs and DMRs by cell type. Percentages reflect the fraction of each genomic region relative to the total, rounded to one decimal place. DMC, differentially methylated cytosine; DMR, differentially methylated region; DMP, differentially methylated promoter.

### Differentially methylated CpG sites, regions and promoters in CD14^+^ monocytes in CVID

In CD14^+^ monocytes, comparison of CVID patients to controls identified 341 DMCs, 3, 057 DMRs, and 268 DMPs (**Figure 2A**). The distribution of hyper- versus hypomethylated DMCs was approximately balanced in CD14^+^ monocytes, whereas DMRs showed a modest predominance of hypomethylation, and approximately 60% of DMPs were hypermethylated. As observed in T-cells, DMCs and DMRs were predominately located in intergenic and intronic regions, with fewer events in promoters and exons (**Figure 2B**).

### Overall methylation patterns and summary of tested regions

Collectively, the CpG analyses in this small discovery cohort indicate numerous methylation differences between CVID and healthy controls. While T-cells were characterized mainly by hypomethylated DMCs and DMRs, monocytes showed a more even distribution of hypo- and hypermethylation across these categories. Chi-square tests nevertheless demonstrated statistically significant differences in hyper- versus hypomethylation between DMCs/DMRs and DMPs in both cell types, with substantially stronger effects in CD3^+^ T-cells (**Supplementary Figure S6**). In both cell types, most epigenetic alterations were captured by DMRs (contiguous genomic regions with coordinated methylation changes) that may function as regulators of gene expression, cell differentiation, and disease processes (21–23, 36). All DMCs, DMRs, and DMPs are provided in **Supplementary Excel File S1**. Volcano plots showing methylation difference versus statistical significance for all tested regions in CD3^+^ T-cells and CD14^+^ monocytes are included in **Supplementary Figure S7**. The plots include approximately 800, 000 regions and are therefore heavily overplotted but are included as an overall summary of the methylation landscape.

### Wnt signalling among the top differentially methylated pathways in CD3^+^ T-cells

We next performed over-representation analysis (ORA) using the Gene Ontology database to identify enriched biological pathways related to CpG methylation. In CD3^+^ T-cells, the most significantly enriched pathways between CVID and healthy controls included T-cell differentiation, actin organisation, and Wnt signalling (**Figure 3A**). Actin, beyond its role in cell motility, reorganizes in T-cells following T-cell receptor activation (37). Wnt signalling encompasses several pathways that regulate cell shape, motility, and differentiation, and play a critical role in T-cell development and function, including interacting with signalling molecules such as JNK and mTOR (36, 38–40). The overlap between selected pathways is shown in **Figure 3B**.

**Figure 3:**
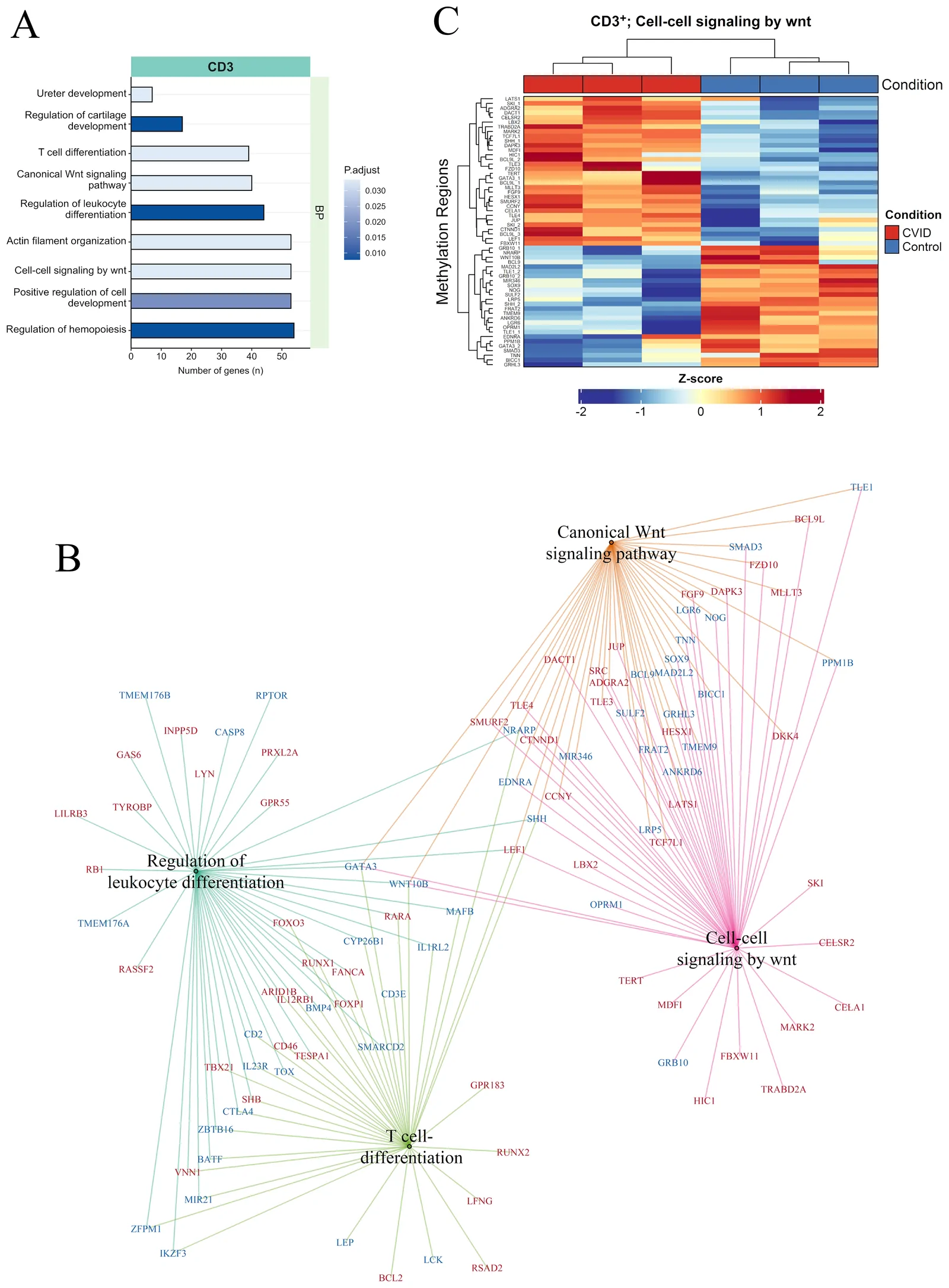
Over-representation analysis of all significant high-frequency DMCs, DMRs, and DMPs in CD3^+^ T-cells, using the Gene Ontology database. **(A)** All enriched pathways found between CVID and healthy controls based on differential methylation in CD3^+^ cells (gene IDs annotated from high-frequency DMCs, DMRs, and DMPs). **(B)** Category gene net plot (cnetplot) of four selected enriched pathways of interest. Enriched pathways appear as central nodes with distinct colours. Significant DMRs associated with the pathways are drawn as lines from the centres, and coloured red if hypermethylated, or blue if hypomethylated. DMRs included in multiple pathways will have multiple connections. **(C)** Heatmap of DMRs related to the enriched “Cell-Cell signalling by wnt” pathway, used to assess whether enrichment is driven by a single sample or reflects coherent patterns across patients.

Because Wnt signalling was among the top enriched pathways in CD3^+^ T-cells, and this pathway seems to be important not only for T-cell function (41), but also for several clinical conditions relevant to CVID, such as autoimmunity and cancer (38), we next examined the robustness of this signal at the CpG level. In CD3^+^ T-cells, none of the CVID patients had identifiable monogenic variants, and unsupervised clustering of methylation levels across 52 Wnt-related DMRs grouped CVID patients and healthy controls separately, with CVID patients showing similar patterns of hyper and hypomethylation across these regions (**Figure 3C**). This supports that the Wnt-related methylation signal in T-cells reflects a shared CVID associated pattern rather than being driven by a single outlier. We also applied unsupervised clustering to DMRs in the T-cell differentiation pathway, which revealed separation of CVID patients and controls and shared methylation patterns among CVID patients (**Supplementary Figure S8A**).

### Pathway-level alterations in CD14^+^ monocytes and impact of a TNFAIP3 variant

CD14^+^ monocytes in CVID compared to controls showed the most significant enrichment for cell adhesion, synaptic/neurological related processes, and actin organisation (**Figure 4A**). Actin in monocytes is crucial for phagocytosis, and altered adhesion pathways could modify monocyte interactions between self and foreign targets (42). The relationship between the pathways and their genes is shown in **Figure 4B**. **Figure 4C** provides a gene-level overview of differential methylation within the actin-related pathways.

**Figure 4:**
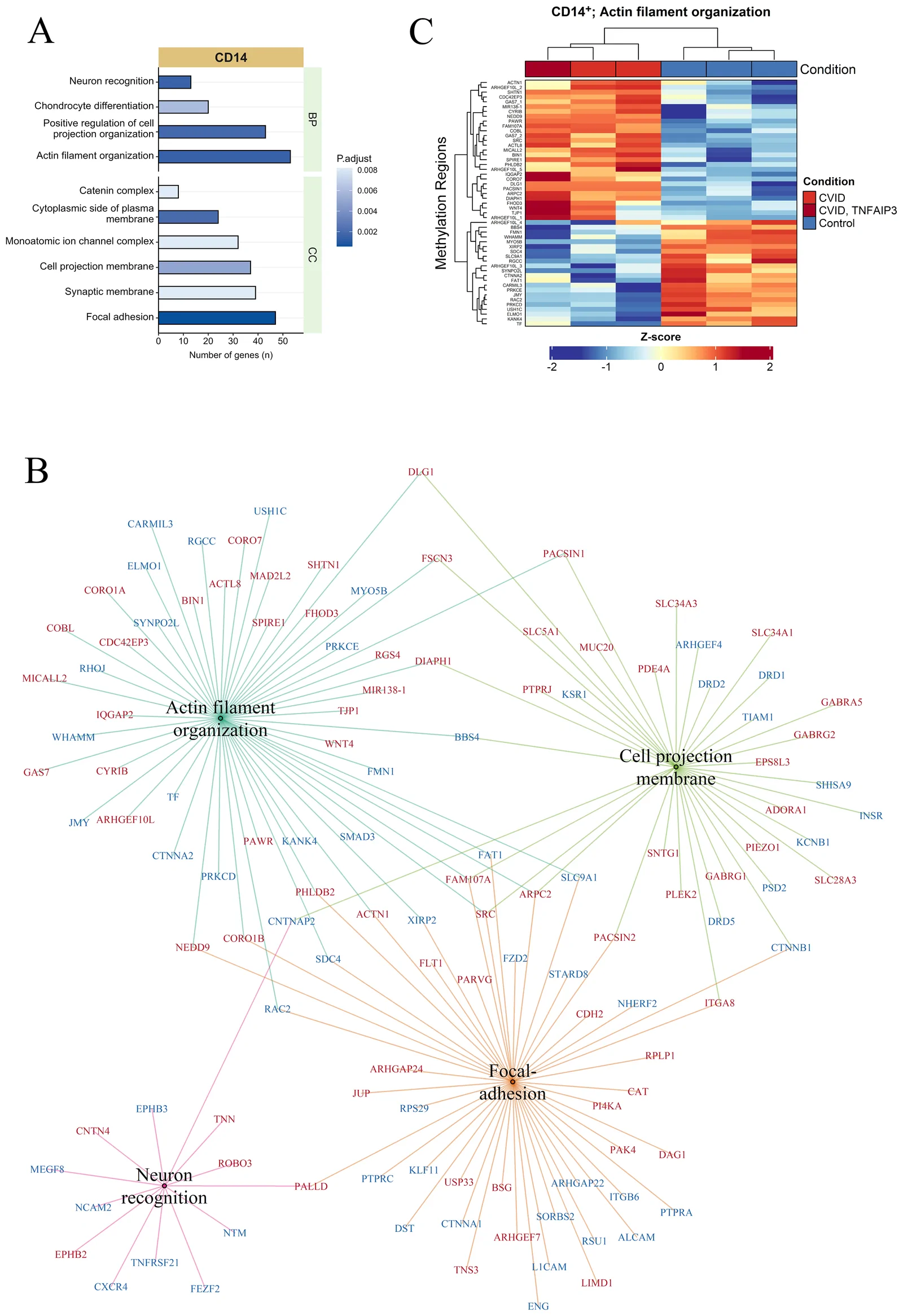
Over-representation analysis of all significant high-frequency DMCs, DMRs, and DMPs in CD14^+^ monocytes, using the Gene Ontology database. **(A)** Top 10 enriched pathways (lowest p-value) between CVID and healthy controls in differential methylation from CD14^+^ cells. **(B)** Category gene net plot (cnetplot) of four selected enriched pathways of interest. Enriched pathways appear as central nodes with distinct colours. Significant DMRs associated with the pathways are drawn as lines from the centres, and coloured red if hypermethylated, or blue if hypomethylated. DMRs included in multiple pathways will have multiple connections. **(C)** Heatmap of DMRs related to the enriched “actin filament organization” pathway, used to assess whether enrichment is driven by a single sample or reflects coherent patterns across patients.

Since CVID is a heterogeneous condition and one of the three CD14^+^ CVID patients carried a TNFAIP3 (A20) variant, we performed a separate pathway analysis of this individual’s monocytes. This analysis revealed hypomethylation in pathways related to Fc gamma receptor-mediated phagocytosis, together with patterns consistent with altered inflammatory/NF-κB related regulation, in line with the known role of TNFAIP3/A20 (11, 34) (**Supplementary Excel File S4**). Because patients with monogenic and non-monogenic CVID can share similar clinical phenotypes (43, 44), we next examined whether this individual disproportionately influenced the pathway level results. As shown in **Figure 4C** and **Supplementary Figures S8B-C**, the TNFAIP3 carrier clustered together with the other CVID patients across all overlapping methylation regions in shared pathways, suggesting that the main CD14^+^ pathway signals are not driven solely by this variant. All ORA results are provided in **Supplementary Excel File S2-4**. Our main conclusions are based on pathway-level findings, and we have taken care to assess their robustness by variant-specific analyses and by demonstrating that the TNFAIP3 carrier clusters with the other CVID patients across the key enriched pathways. The inclusion of this patient is still a limitation of our monocyte data.

### Altered plasma levels of Wnt-related proteins in an extended CVID cohort

Given that the Wnt-related methylation signal in CD3^+^ T-cells were among the most prominent signals, and in light of the recognised importance of this pathway in T-cell function and autoimmunity (38, 41), we next examined whether Wnt pathway involvement was also reflected at the systemic protein level. We address this we measured five secreted Wnt modulators in plasma from a larger cohort comprising 60 CVID patients (37 women, 62%; mean age 43 years) and 30 healthy controls (18 women, 60%; mean age 45 years) (**Table 2**). The Wnt modulators included four extracellular antagonists (DKK1, DKK3, sFRP3 and WIF), and one extracellular agonist (RSPO3).

In T cell and monocyte methylation analyses, we only included patients from the “complicated CVID” subgroup. In the plasma cohort 47 (78%) had autoimmune or inflammatory complications (“CVID complicated”) and 13 had “infection only”. Clinical characteristics are listed in **Table 2**, and a brief description of the proteins and their role in Wnt signalling are provided in **Supplementary Table S1**.

When comparing the entire CVID cohort with healthy controls, levels of the Wnt antagonists DKK1 and DKK3 were significantly lower in CVID, whereas the Wnt agonist RSPO3 was higher (**Figure 5A**, **Supplementary Excel File S5**). Plasma levels of sFRP3 and WIF did not differ between CVID patients and healthy controls.

**Figure 5:**
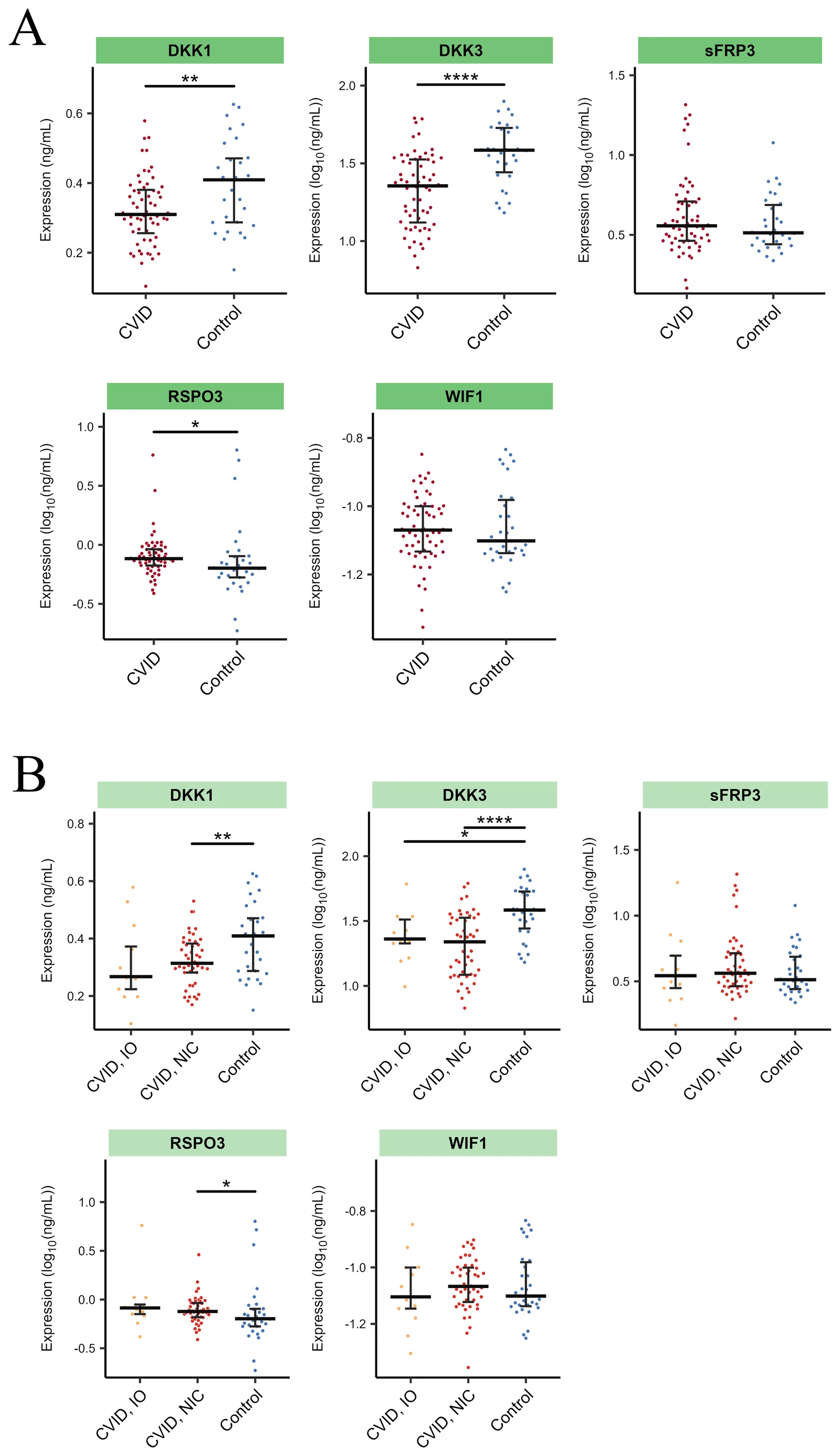
Plasma levels of five Wnt-related proteins in CVID compared to healthy controls. **(A)** All CVID patients (n = 60) versus healthy controls (n = 30). **(B)** CVID patients with infections only (IO, n = 13) and CVID patterns with non-infectious complications (NIC, n = 47) versus healthy controls (n = 30). DKK1, DKK3, sFRP3 and WIF are extracellular Wnt antagonists; RSPO3 is an extracellular Wnt agonist. Most analytes showed skewed distributions and are therefore plotted on a log10 scale on the y-axis; only approximately normally distributed data are shown on a linear scale. Group differences were assessed using unpaired t-tests for normally distributed data, and t-tests or Mann-Whitney U test on log10-transformed values for moderately skewed data. **** = p < 0.0001, *** = p < 0.001, ** = p < 0.01, * = p < 0.05.

We then compared plasma levels of Wnt modulators between the two clinical subgroups CVID patients, i.e., “CVID infection only” and “CVID complicated”. For DKK3, levels differed significantly from controls in both groups of CVID patients with lower levels in both sub-groups (**Figure 5B**, **Supplementary Excel File S5**). For DKK1 and RSPO3, mean levels in CVID with infection only were lower (DKK1) and higher (RSPO3), respectively, than in CVID with autoimmune and inflammatory complications, but these differences did not reach statistical significance (p-values 0.08 and 0.07, respectively), consistent with limited power in the small CVID “infection only” subgroup.

### Wnt-related RNA expression across independent CVID transcriptomic studies

To further validate our findings of altered Wnt signalling in CVID, we finally examined the Wnt-related transcriptome expression in publicly available RNA datasets from T-cells or PBMC in three previous CVID studies (15, 31, 32). In these datasets, 60–101 significant Wnt-related genes were identified per study (**Supplementary Excel File S6**), and the overlap between studies is summarised in **Figure 6A**.

**Figure 6:**
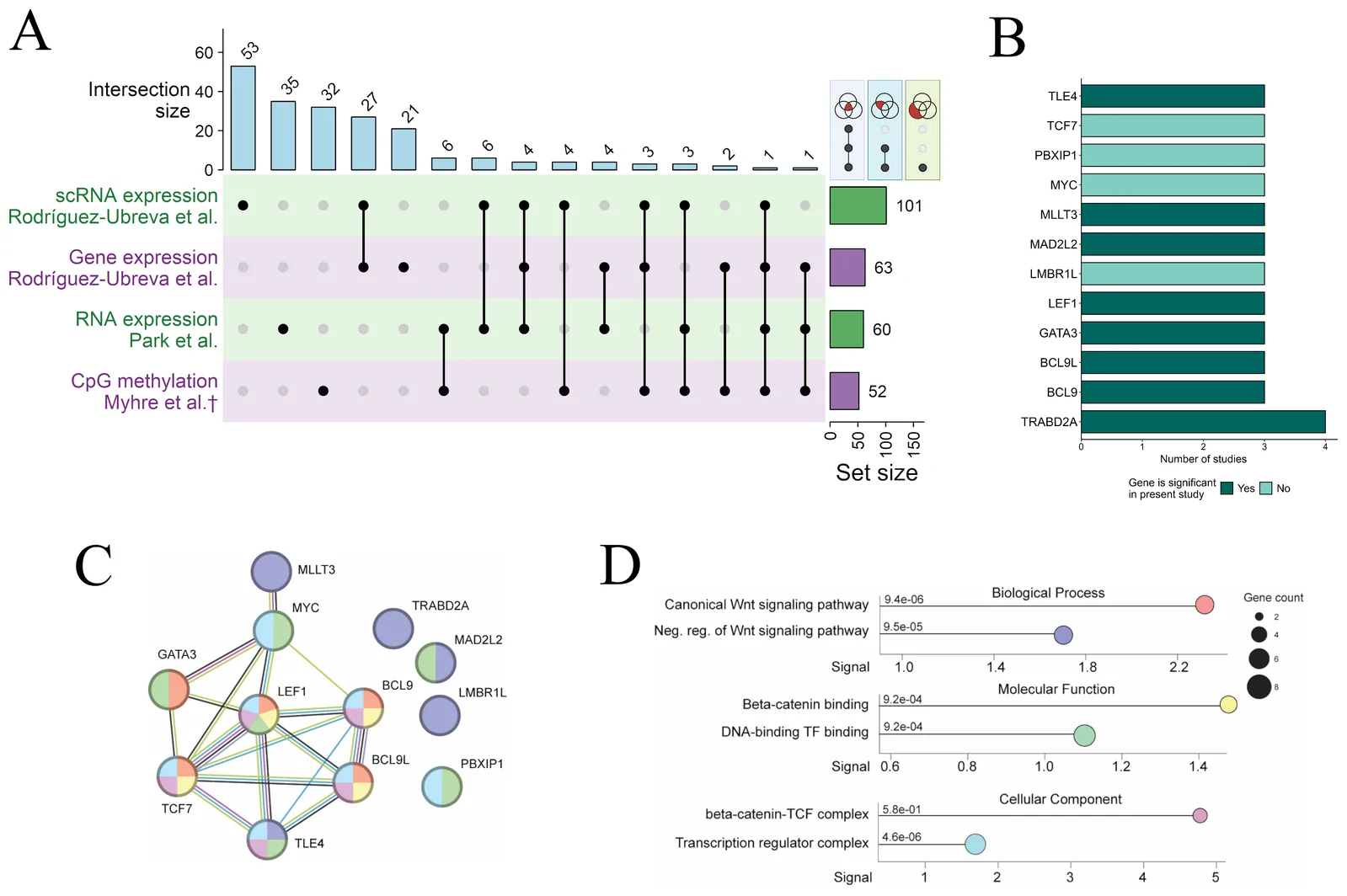
Wnt-related gene expression in other studies compared to present methylation study. RNA data were obtained from whole blood (31), CD4^+^ and CD8^+^ T-cells (32), and T-cells (15). **(A)** UpSet plot showing the number and overlap of significant Wnt-related genes across the published studies and our CpG methylation dataset. For each dataset, Wnt related RNAs (in the RNA studies) or CpGs (present study, Myhre et al.) were mapped to gene IDs and overlap is shown at gene level. The total number of Wnt-related genes from each dataset is shown as barplots to the right (altering between green and purple). The schematic in the upper right illustrates how to interpret the dots: a single black dot represents genes that are unique to one dataset, and connected dots represent genes shared between datasets. **(B)** All significant genes that are present in at least three of the four datasets. **(C)** Interactions between genes shown in Figure B Genes are coloured according to the pathways in which they are involved in panel D by the circle colours; genes involved in multiple pathways are multi-coloured. **(D)** Significantly enriched pathways for the genes from panel B, using the Gene Ontology database. Higher signal indicates stronger enrichment, calculated as the log10 ratio of observed genes divided by expected genes. † = present study.

Twelve Wnt-related transcripts were detected in at least three of the four datasets (**Figure 6B**). Several of these Wnt-related RNAs were also shown to be involved in transcription factor-related pathways (**Figures 6C, D**) in line with Wnt-mediated transcriptional regulation being driven by β-catenin-T-cell factor complexes (45). Together with our methylation and plasma protein data, these findings indicate that Wnt-related alterations are recurrent across independent CVID cohorts and across multiple molecular layers.

## Discussion

To the best of our knowledge, this is the first study to explore genome-wide CpG methylation in purified T-cells and monocytes in CVID. We report two main novel observations: first, that CD3^+^ T-cells and CD14^+^ monocytes show evidence of cell type-specific epigenetic alterations; and second, that Wnt signalling emerges as a recurrently altered pathway, supported across DNA methylation data, circulating Wnt-related proteins and independent transcriptomic datasets. Taken together, these findings point to previously unrecognized epigenetic and Wnt-related mechanisms that may contribute to the immunological phenotype of CVID.

Previous epigenetic work in CVID is limited and has mainly focused on B-cells and duodenal tissue (14–18). In parallel, large genetic studies have identified monogenic causes in only a subset of patients, leaving the majority without a clear genetic explanation (11). This has raised the possibility that epigenetic mechanisms in other immune cell types, such as T-cells and monocytes, may contribute to disease pathogenesis. Although our discovery cohort is small, whole-genome bisulphite sequencing atlases in other tissues indicate that methylation profiles within a given cell type are highly reproducible across individuals when at least three samples are analysed (46). Our data add an important layer by showing CpG methylation differences in CD3^+^ T-cells and CD14^+^ monocytes from CVID patients with inflammatory complications. The predominance of hypomethylated DMCs/DMRs in T-cells is compatible with an “open” chromatin landscape and activated or poised states, which fits well with reports of skewed Th1 responses, reduced Treg frequencies and chronic T-cell activation in CVID (7–9). In monocytes, a more balanced pattern of hypo- and hypermethylation may reflect that epigenetic modifications in these cells contribute to the coexistence of pro- and anti-inflammatory programmes, consistent with increased cytokine production and altered innate responses described in this disease (10–12).

A novel finding of this study is the involvement of Wnt signalling across several molecular levels. Wnt is increasingly recognized as a key regulator of T-cell differentiation, memory formation and tolerance, as well as of haematopoietic stem cell and progenitor function (38). In experimental models, canonical and non-canonical Wnt pathways influence T-cell survival, the balance between effector and regulatory subsets, and the propensity for exhaustion, partly through crosstalk with mTOR and NF-κB (38, 39, 47–49). In this context, the prominent Wnt signal in our epigenetic data is notable. Importantly, Wnt pathway alterations have also been reported in duodenal tissue from CVID enteropathy, where RNA sequencing has shown downregulation of Wnt signalling in villous atrophy lesions, suggesting epithelial involvement (50). Pathway analyses identified Wnt among the top differentially methylated pathways in CD3^+^ T-cells, alongside immune pathways such as T-cell differentiation and actin organisation. Clustering of Wnt related DMRs clearly separated CVID from controls and showed coherent patterns across patients, suggesting a shared disease associated imprint rather than random noise. That this signature emerged in purified T-cells, and in the absence of known monogenic variants in the T-cell cohort, is compatible with the notion that Wnt-related methylation changes could be a genuine feature of CVID T-cell biology. The Wnt signal in the epigenetic analyses was further supported at the transcriptomic and protein levels.

When measuring selected Wnt proteins in plasma from CVID patients and healthy controls, we found that CVID patients had reduced plasma levels of DKK1 and DKK3, inhibitors of the canonical Wnt/β-catenin signalling pathway, and increased levels of RSPO3, an activator of this pathway, potentially suggesting a net enhancement of canonical Wnt activation in CVID. Subgroup analyses showed no significant differences between “CVID complicated” and “CVID infection only”, but these analyses were weakened by the low number of patients in the “CVID infection only” group, consistent with limited statistical power. One could speculate that these patterns may suggest that the disturbed Wnt profile is related to the immunodeficiency per se, but the available data do not allow any firm conclusion. An alternative interpretation is that these Wnt-related changes primarily reflect chronic immune dysregulation and inflammatory complications rather than CVID (i.e. B-cell deficiency) per se, and larger, well-characterised cohorts will be required to disentangle pathway signatures related to complicated CVID from those related to CVID more broadly.

While plasma measurements cannot pinpoint cellular sources, this pattern is consistent with an overall shift towards enhanced Wnt activity in CVID. Reanalysis of three independent CVID transcriptomic datasets, showing recurrent differential expression of Wnt-related genes with overlap to our methylation findings, provides an additional, external line of support. Overall, these observations suggest that Wnt signalling is not an incidental finding confined to a single dataset, but a recurrent pathway in CVID.

From a mechanistic standpoint, altered Wnt activity might contribute not only to autoimmune and inflammatory complications, but also to more fundamental features of CVID, such as impaired regulatory T-cell function (47). Moreover, β-catenin, as an important mediator in canonical Wnt signalling, has been reported to be dispensable for normal B-cell development and function, and is therefore unlikely to underlie the B-cell maturation defect seen in CVID (51). Functional studies will be required to test these hypotheses, and to determine whether Wnt related changes have direct causal roles in CVID pathogenesis. Of interest, the Wnt signalling pathway has emerged as a novel target for therapy in various disorders (38), including approaches targeting RSPO3, which was upregulated in the present CVID plasma cohort (52).

Wnt has not previously been systematically explored in CVID, and several key Wnt modulators are measurable in plasma, which made this pathway particularly suitable for further, hypothesis-driven investigation. This does not imply that Wnt is more important than other enriched pathways such as actin organisation or T-cell differentiation. Our plasma protein analyses and the Wnt-focused re-analysis of public RNA datasets should therefore be viewed as targeted follow-up of one underexplored pathway, and we acknowledge that this *post hoc* focus introduces a risk of confirmation bias compared with an unbiased pathway screen. Overall, our findings highlight Wnt signalling as a recurrent and mechanistically attractive axis alongside other pathways in CVID, without establishing it as the only or uniquely dominant pathway.

In CD14^+^ monocytes, exploratory pathway analysis highlighted processes related to cell adhesion, actin organisation and synaptic/neurological signalling. These pathways are relevant for monocyte trafficking, phagocytosis and immune synapse formation, and are in line with reports suggesting that monocytes/macrophages contribute to tissue inflammation and autoimmunity in CVID (10–12). Interpretation of the monocyte data is, however, complicated by the presence of a TNFAIP3 (A20) variant in one of the three CVID patients. Our separate pathway analyses of this individual’s monocytes indicated hypomethylation in pathways related to Fc gamma receptor-mediated phagocytosis and patterns compatible with altered NF-κB-related regulation, in line with the known biology of TNFAIP3 (34) and supporting the validity of our analytical approach. As the heatmaps suggest, this patient does not appear as a clear outlier for the main CD14^+^ pathway findings, illustrating how patients with a defined monogenic variant and those without an identified genetic cause can converge on similar pathway level and epigenetic changes. This resonates with the ongoing discussion about whether patients with identified monogenic defects should still be classified under CVID or considered as separate entities (11, 53).

Important limitations must be acknowledged. First, the discovery cohort is very small, which makes WGBS-based identification of differentially methylated CpGs and regions susceptible to inter-individual variability, batch effects and reduced statistical power. The discovery cohort did not include cells from CVID patients with “infection only”, and the monocyte data are further complicated by the presence of a TNFAIP3 variant in one of the three CVID patients, whereas the CD3^+^ T-cell cohort comprised CVID patients without identified monogenic defects. Although our clustering and variant-specific pathway analyses indicate that the main actin/adhesion-related signals are not solely driven by this carrier, the very small sample size precludes robust sensitivity analyses excluding this patient, and the monocyte results should therefore be interpreted with particular caution. Furthermore, the epigenetic analyses were performed on bulk CD3^+^ T-cells and CD14^+^ monocytes and did not include cellular subsets. An additional limitation is the substantial time gap between the discovery and validation cohorts. Cohort composition, genetic work-up and unmeasured secular changes in CVID care may therefore differ over time. However, the core principles of immunoglobulin replacement therapy have remained comparable across this period, and patients receiving immunosuppressive or immunomodulatory drugs were excluded from both cohorts, so major changes in such therapies are unlikely to have directly influenced our results. The cross-sectional design, the lack of functional assays of Wnt pathway activation in CVID cells (e.g. reporter assays or downstream target gene readouts), the lack of adjustment for kidney function and bone metabolism, both of which could influence levels of Wnt modulators (54), the absence of histone modification data, which are important mediators of epigenetic regulation, further limits our ability to infer causality from the observed methylation and plasma protein patterns. Taken together, these limitations mean that the epigenetic results should be regarded as exploratory and hypothesis-generating rather than definitive.

## Conclusions

In conclusion, this exploratory study provides initial evidence that CVID is associated with cell type-specific epigenetic remodelling in T-cells and monocytes, and identifies Wnt signalling as a recurrently altered pathway across DNA methylation, circulating proteins, and independent transcriptomic datasets. In the context of existing literature, these findings support an emerging concept of CVID as a disorder of broader immune reprogramming beyond B-cell dysfunction and highlight Wnt signalling and related regulatory networks as promising targets for future mechanistic and, potentially, therapeutic exploration. However, these issues need to be more thoroughly studied in larger cohorts with prospective follow-up and mechanistic experiments.

## Data Availability

Availability of data and materials. The original datasets are not publicly available due to Norwegian legislation regarding data protection, but are available from the corresponding author (SFJ), upon reasonable request.
