# Supplementary material for: T-cell and monocyte DNA methylation and Wnt dysregulation in common variable immunodeficiency: an exploratory study
Source: Front Immunol. 2026 Oct 1;17:1931297. doi: 10.3389/fimmu.2026.1931297 (PMC13668603; doi:10.3389/fimmu.2026.1931297)
Supplement: Supplementary file 1 [file SupplementaryFile1.pdf]

Supplementary Information

T-Cell and Monocyte DNA Methylation and Wnt Dysregulation in Common Variable Immunodeficiency; an exploratory study

Vegard Myhre MSc[1,2], Mingyi Yang PhD[3,4], Mari Kaarbø PhD[3], Mai Sasaki Aanensen Fraz MD[1,2,5], Annika E. Michelsen PhD[1,2], Børre Fevang MD, PhD[2,5], Pål Aukrust MD, PhD[1,2,5], Thor Ueland PhD[1,2,6], Magnar Bjørås Cand Scient[3,7,8], Silje Fjellgård Jørgensen MD, PhD[1,5*]

*Corresponding author: s.f.jorgensen@medisin.uio.no

1. Research Institute of Internal Medicine, Division of Surgery and Specialized Medicine, Oslo University Hospital, Oslo, Norway.
2. Institute of Clinical Medicine, University of Oslo, Oslo, Norway.
3. Department of Microbiology, Oslo University Hospital, Oslo, Norway.
4. Department of Medical Biochemistry, Oslo University Hospital, Oslo, Norway.
5. Section of Clinical Immunology and Infectious Diseases, Oslo University Hospital, Rikshospitalet, Oslo, Norway.
6. Faculty of Health Sciences, K.G. Jebsen TREC, University of Tromsø, Tromsø, Norway.
7. Department of Clinical and Molecular Medicine, Norwegian University of Science and Technology, NTNU, Trondheim, Norway.
8. Department of Microbiology, University of Oslo, Oslo, Norway.

Supplementary Information

Supplementary Materials and Methods

Supplementary Figures

Supplementary Figure S1: Overall bioinformatic workflow.

Supplementary Figure S2: CpG read coverage for $CD3^+$.

Supplementary Figure S3: CpG read coverage for $CD14^+$.

Supplementary Figure S4: CpG sites before and after filtering, and maximum site numbers for DMCs, DMRs, and DMPs.

Supplementary Figure S5: Heatmaps of methylation regions and DMRs

Supplementary Figure S6: Chi-square test of independence in hypo-/hypermethylation ratios of DMCs, DMRs, and DMPs

Supplementary Figure S7: Vulcano-plots of all methylation regions

Supplementary Figure S8: Additional heatmaps from over-representation analysis

Supplementary Tables

Supplementary Table S1: UniProt function for Wnt-related proteins.

Supplementary Excel Files (separate files)

Supplementary Excel File S1: DMCs, DMRs, DMPs, and high-frequency DMCs in $CD3^+$ and $CD14^+$.

Supplementary Excel File S2: Over-representation analysis of differential methylation, CVID compared to healthy controls. $CD3^+$

Supplementary Excel File S3: Over-representation analysis of differential methylation, CVID compared to healthy controls. $CD14^+$

Supplementary Excel File S4: Over-representation analysis of differential methylation, CVID patient with TNFAIP3 variant compared to CVID. $CD14^+$

Supplementary Excel File S5: Statistical results for Wnt expression.

Supplementary Excel File S6: Wnt expression in previous studies.

Supplementary Materials and Methods

Whole-genome bisulphite sequencing (WGBS) analysis

WGBS was performed by Admera Health, NJ, USA with a few modifications as previously described (1). The samples were lysed in RLT buffer with β-mercaptoethanol and homogenized using a QIAshredder (Qiagen). Genomic DNA was eluted in molecular grade H2O and stored at -20°C. Genomic DNA was quantified with Qubit 2.0 DNA HS Assay (ThermoFisher, Massachusetts, USA) and quality assessed by Tapestation genomic DNA Assay (Agilent Technologies, California, USA).

Genomic DNA was bisulphite converted with the EZ DNA Methylation-Gold Kit (Zymo Research, California, USA) following manufacturer's protocol. Library preparation was then performed using Accel-NGS®Methyl-Seq DNA Library kit (Swift Biosciences, Michigan, USA) per manufacturer's recommendations. Final libraries were assessed by APA SYBR® FAST qPCR with QuantStudio ® 5 System (Applied Biosystems, California, USA) and quality was assessed by TapeStation HSD1000 ScreenTape (Agilent Technologies Inc., California, USA). Equimolar pooling of libraries was performed based on QC values. Samples were sequenced on an Illumina® HiSeq (Illumina, California, USA) with a read length of 150 bp and 300 M (millions) paired-end (PE) reads per sample.

The initial processing data of genome-wide 5mC counts was generated by Admera Health. Briefly, the following steps were included: the raw data of FASTQ files were cleaned by Trim Galore (v0.6.5) and subjected to quality control by FastQC (v0.11.9). The cleaned WGBS data (about 300 M PE reads per sample) was aligned to the Hg38 human reference genome, followed by extraction of genome-wide 5mC in CG, CHG and CHH context separately using Bowtie2 (v2.4.1) and Bismark (v0.22.3).

CpG analysis in methylKit

The methylKit (v1.34.0) package was used to analyse CpG methylation across both cell types. FilterByCoverage() was used with default settings, except “lo.count” was set to 5, for initial filtering. The filtered data was used for three separate calculations.

First, DMCs were identified using unite() with argument “destrand = FALSE”, followed by calculateDiffMeth() with parameters “test = Chisq”, “overdispersion =”MN”, adjust = “BH”. DMCs were then defined using a cutoff of Benjamini-Hochberg adjusted p-value < 0.05, and a mean methylation difference of at least |10%|.

Second, the data was used to identify DMRs. The data was used with tileMethylCounts() set with “win.size” and “step.increase” to 1000, and “cov.bases = 3”. This was followed unite() with “destrand = TRUE”, and then calculateDiffMeth() with similar parameters as for DMCs. DMRs were then identified using a cutoff of BH-adjusted p-value < 0.05, and a mean methylation difference of at least |10%|.

Third, the data was used to identify DMPs. A refseq HG38 BED-file was read using readTranscriptFeatures() with up- and down flanks set to 1000, which defined promoter regions to TSS +- 1000 bp. The promoter regions were annotated to the data using regionCounts() with “cov.bases = 3”. This was followed unite() with “destrand = TRUE”, and calculateDiffMeth() with similar parameters as for DMCs. DMPs were then identified after applying a threshold of BH-adjusted p-value < 0.05, and a mean methylation difference of at least |10%|.

We defined a subset of DMCs as high-frequency DMCs, defined as DMCs with at least two DMCs at TSS +- 10kb, could be annotated to a gene, and with a mean methylation difference of at least |25%|.

Overall, for $CD3^+$, 1,649,218 sites were available for differently methylated cytosine (DMC) analysis, 805,722 regions for differently methylated regions (DMR) analysis, and 72,482 promoters for differently methylated promoter (DMP) analysis. In $CD14^+$, there were available 1,654,414 cytosines, 776,336 regions, and 72,240 promoters, respectively, for DMC, DMR, and DMP analysis.

Gene and DNA region annotations

Genes and DNA regions were annotated by using Homo sapiens genome assembly 38(2). Promoter regions were defined as transcription start site +/- 1kb. Intergenic regions were defined as regions that were not in promoters, exons, or introns.

General R-packages used

The tidyverse (v2.0.0) and here (v1.0.1) packages were used in general during the analysis. Rstatix (v0.7.3) was used for Fisher's exact, Chi-squared, Kruskal-Wallis, and t-tests. PCA was performed by using the stats (v3.6.2) package.

Over-representation analysis

The packages clusterProfiler (v4.6.2), AnnotationDbi (v1.64.1) and org.Hs.eg.db (v3.18.0) were used for Over-representation analysis (ORA), using the Gene Ontology (GO) database. P-values were corrected using Benjamini-Hochberg.

Additional analysis of TNFAIP3 variant in a CVID patient

Methylation percentages from regions in $CD14^+$ were converted to Z-scores including only CVID patients (i.e. excluding the healthy controls). We identified the regions in the CVID patient with the TNFAIP3 variant that showed a Z-score higher than at least |1|, and then selected the top 10% by absolute value. We then performed ORA and compared the significantly enriched pathways with the pathways found between the CVID group as a whole

compared to healthy controls. Since the annotated genes that drive a pathway to significance are known in an ORA, we then compared the annotated genes in shared significant pathways to DMRs to investigate if the CVID patient with the TNFAIP3 variant was a driver for the whole CVID group.

Analysis of Wnt proteins

Five Wnt related proteins from blood plasma were measured in an extended cohort comprising healthy controls and CVID patients. Among the CVID patients, two clinical subgroups were defined: those with "infection only" and those with non-infectious complications. For the primary analyses, all CVID patients were first considered as one combined group ("CVID all") and compared with healthy controls. In secondary analyses, the two CVID subgroups ("infection only" and "non-infectious complications") were then compared with each other and with healthy controls. Our analysis proceeded in three steps. First, we assessed the distribution of each of the five proteins in all groups using the Shapiro-Wilk test of normality. DKK1 was the only protein with no evidence of skewed distribution in any group and was therefore analysed on the original scale. The other four proteins were $\log_{10}$ transformed. Second, a new Shapiro-Wilk test was performed on the transformed data for each group and proteins. Third, for group comparison, we used Student's t-test if t all relevant groups for a given protein were normally distributed according to the second Shapiro-Wilk test; otherwise, the Mann-Whitney U test was applied. No p-value correction for multiple testing was applied.

Wnt expression evaluation from previous studies

Wnt expression evaluation was performed by comparing our Wnt-related genes showing differential methylation to mRNA expressions from three CVID studies.

A study by Park et al. (3) analysed RNA from whole blood. We downloaded the training and test sets using the getGEO() function from the GEOquery (v2.78.0) R-package, with GEO = "GSE51406". We merged the two datasets and excluded CVID patients without complications, for a final sample size of CVID with complications (n = 47) and healthy controls (n = 36). Shapiro-Wilk test of normality was used to identify normally distributed RNA expression values in CVID and healthy controls. A t-test was used if both groups showed a normal distribution, and if not, the Mann-Whitney U-test was used instead. No p-value correction was applied.

A study by Rodríguez-Ubreva et al. (4) analysed single cell RNA from peripheral blood mononuclear cells in COVID-19 CVID patients. We downloaded the "supplementary data 3" dataset, which included differently expressed genes per cell compartment, and filtered for data at baseline before COVID-19 infection. We then merged significant genes from the $CD4^+$ and $CD8^+$ cells.

A study by Rodríguez-Ubreva et al. (5) investigated differences between monozygotic twins, in which one twin developed CVID while the other did not. We downloaded the "supplementary data 7", which included differently expressed genes in different cell compartments between the twins. We then merged all differently expressed genes in T cells.

Figures

Figures were made using ggplot2 (v3.5.0), ggpubr (v0.6.0), and ggh4x (v0.2.8). We used palettes from RColorBrewer (v1.1.3) with more colour-blind friendly colours. The UpSet plot was made using CompexHeatmap (v2.24.1), and with the "distinct" setting.

Manuscript preparation

The manuscript was written in Rstudio using rmarkdown (v2.30). Tables were made using flextable (v0.9.10).

Supplementary Figures

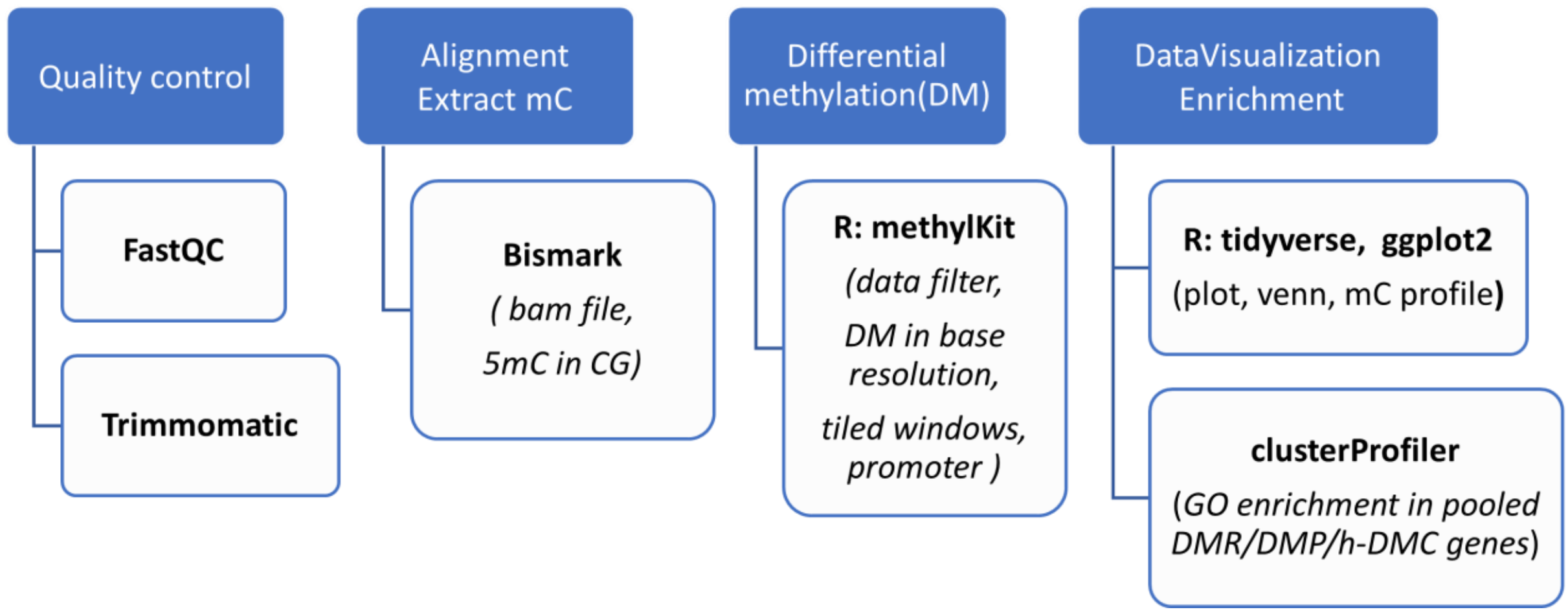

Supplementary Figure S1: Overall bioinformatic workflow. *Quality control for the raw reads was performed with FastQC. The adapters and low-quality reads were removed with Trimmomatic tool. The paired reads were further aligned against reference genome, the counts of 5mC and unmethylated C in each base were extracted in context of CG by Bismark package. The methylKit tool was used for computing differential methylation in base resolution (DMC) and region (DMR) via tiled-windows. The data was visualised by R packages.*

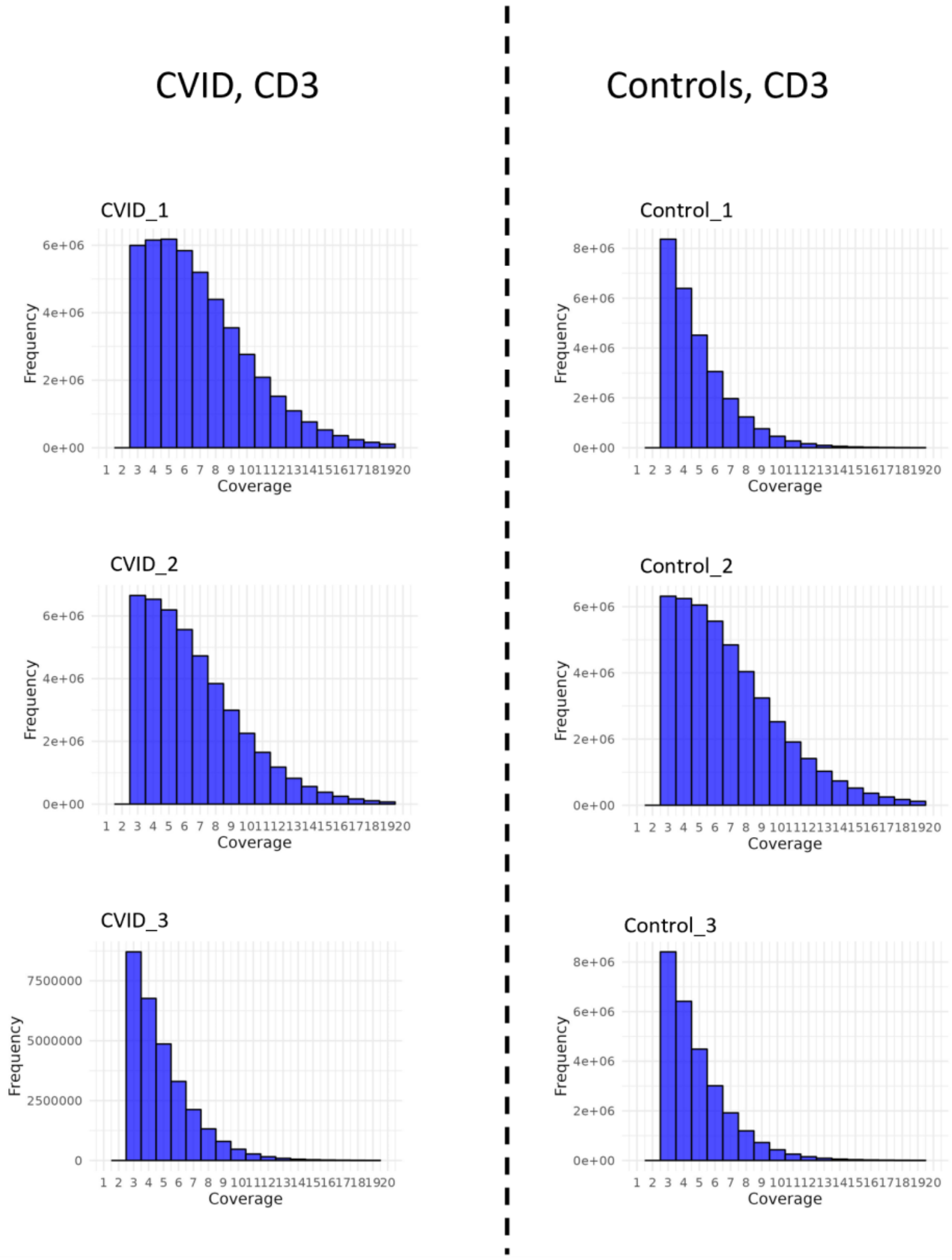

Supplementary Figure S2: Read depth for CD3^{+}. *Read depth for all CpG sites covered by whole genome bisulphite sequencing.*

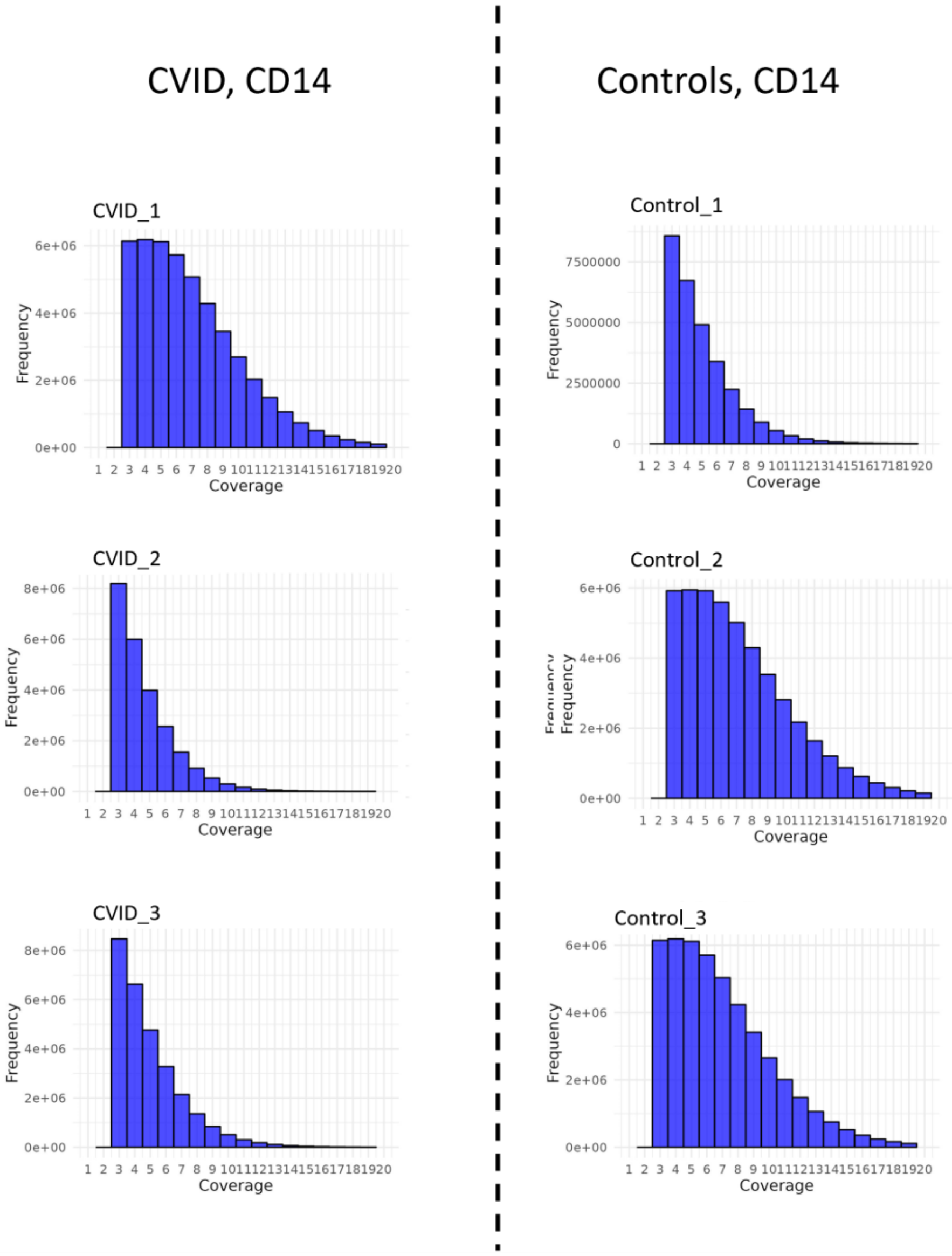

Supplementary Figure S3: Read depth for CD14^{+}. *Read depth for all CpG sites covered by whole genome bisulphite sequencing.*

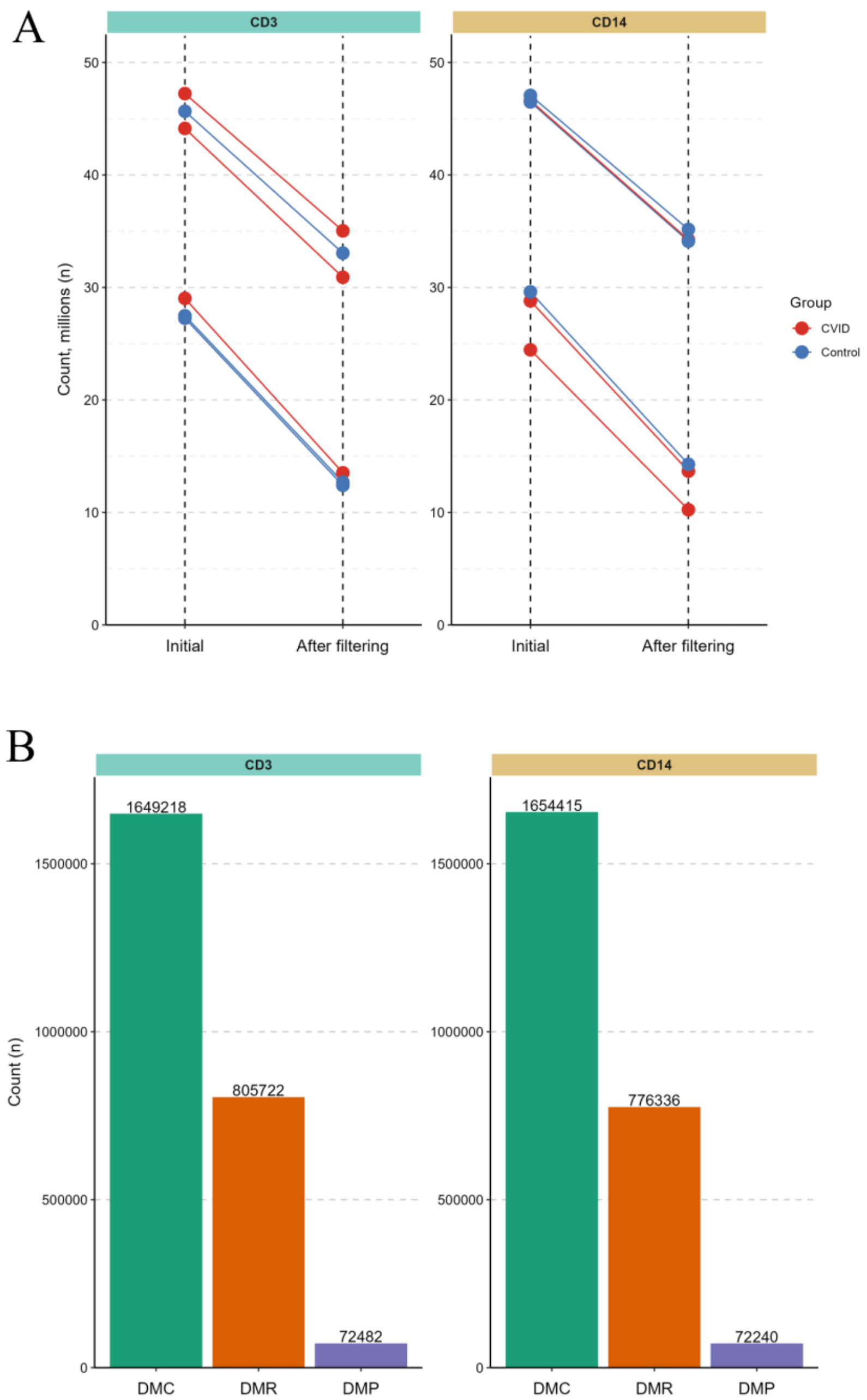

Supplementary Figure S4: Overall methylation coverage. A) *CpG site coverage for each sample before and after read depth filtering.* ***B)*** *Total number of potential DMCs, DMRs, and DMPs in this analysis. DMC, differentially methylated cytosine; DMR, differentially methylated region; DMP, differentially methylated promoter.*

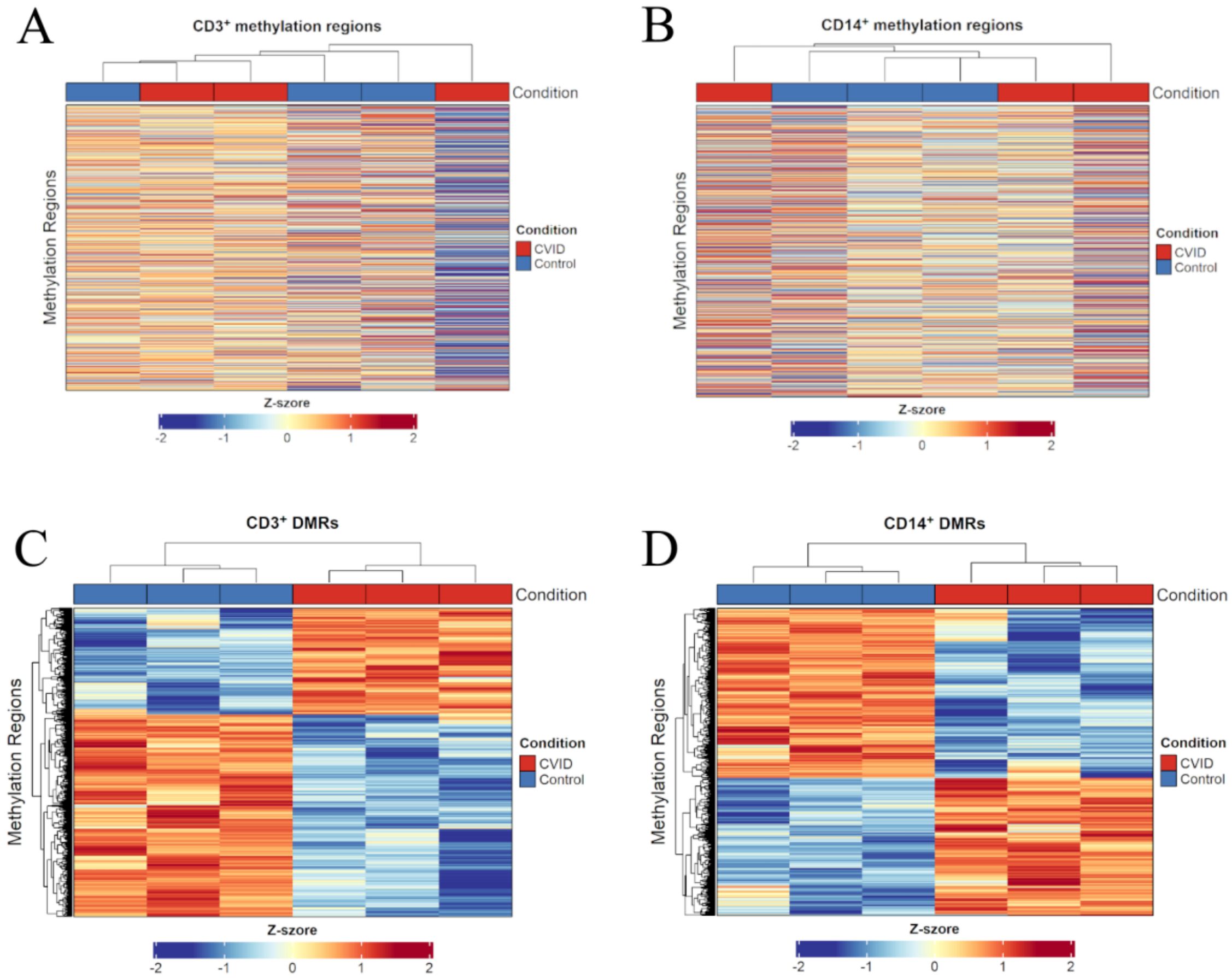

Supplementary Figure S5: Heatmaps of methylation regions and differentially methylated regions (DMRs).
A) *Heatmap of all methylation regions (n = 805,722) analysed in CD3⁺ cells. Top dendrogram show unsupervised clustering of all samples. No left dendrogram could be made due to the number of methylation regions included.* ***B)*** *Heatmap of all methylation regions (n = 776,336) analysed in CD14⁺ cells. Top dendrogram show unsupervised clustering of all samples. No left dendrogram could be made due to the number of methylation regions included.* ***C)*** *Heatmap of all significant DMRs (n = 3,727) identified in CD3⁺ cells between CVID and healthy controls.* ***D)*** *Heatmap of all significant DMRs (n = 3,057) identified in CD14⁺ cells between CVID and healthy controls.*

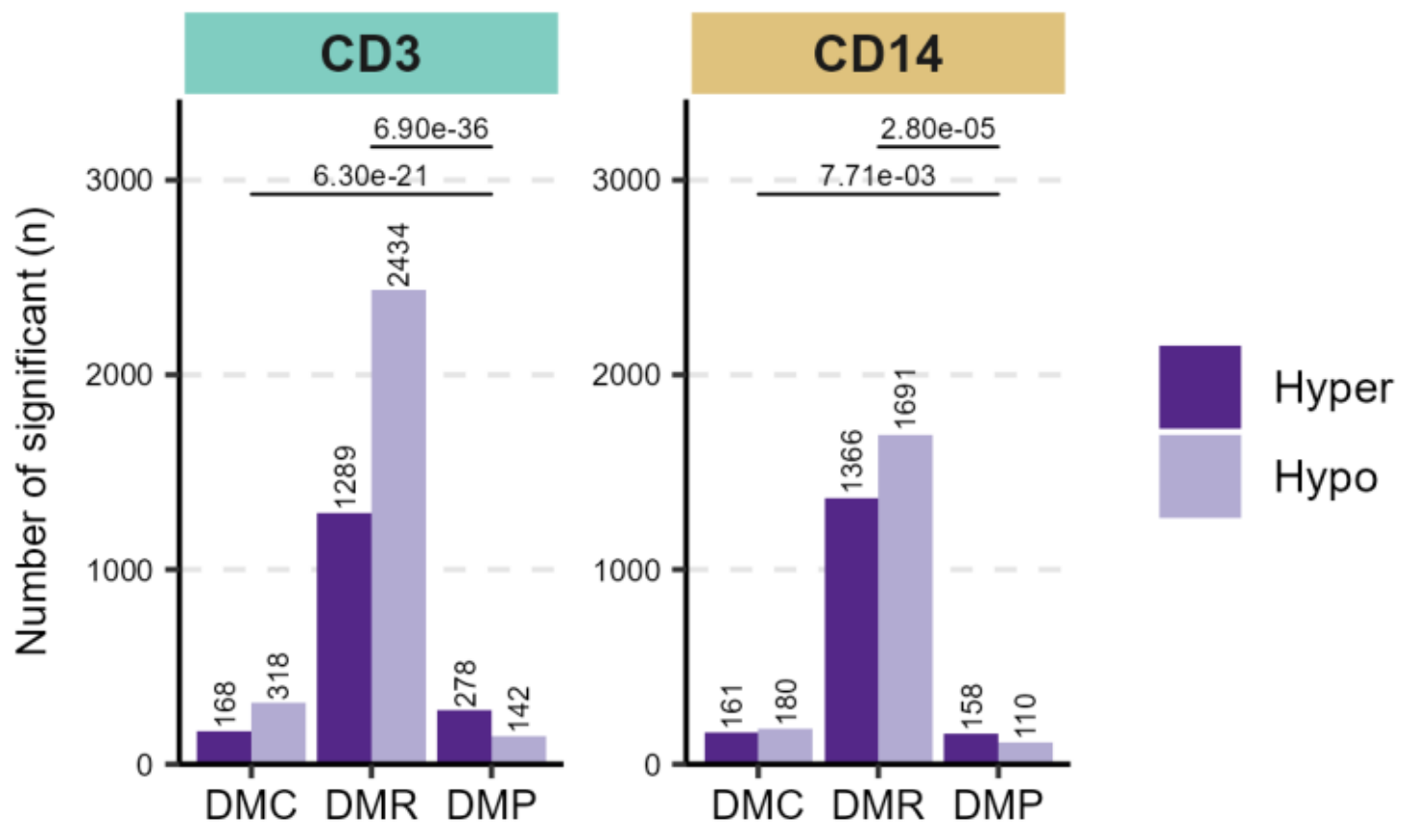

Supplementary Figure S6: Chi-square test of independence in hypo-/hypermethylation ratios of DMCs, DMRs, and DMPs in CD3^{+} and CD14^{+} cells. *Identification of DMCs, DMRs, and DMPs in CVID compared to healthy controls revealed potential differences in hypo-/hypermethylation between the differential methylation types. The ratios were then tested using the Chi-square test of independence, and the p-values were corrected using Benjamini-Hochberg. All p-values are displayed above the horizontal black lines, representing a pairwise comparison. DMC, differently methylated cytosine; DMR, differently methylated region; DMP, differently methylated promoter.*

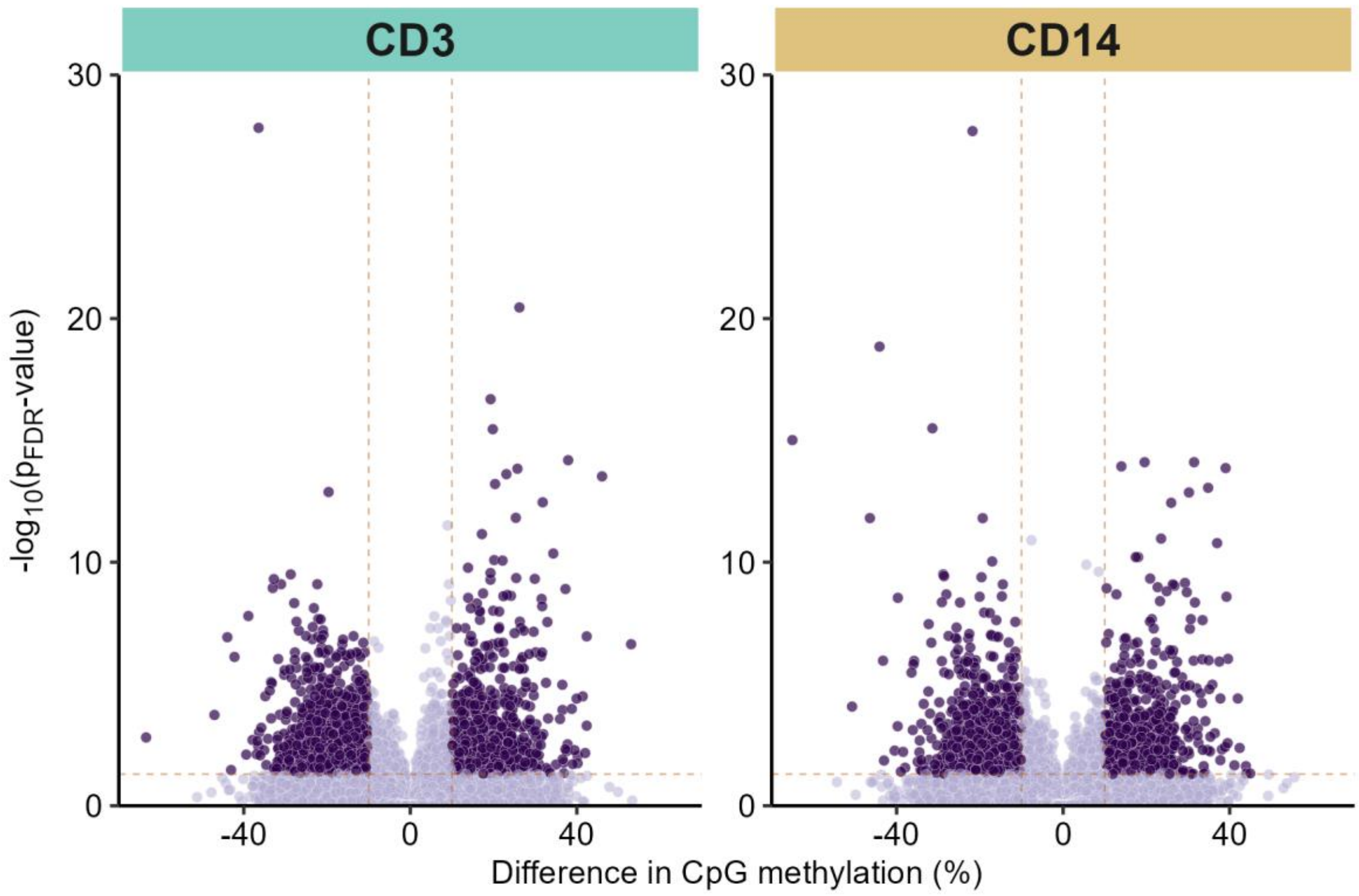

Supplementary Figure S7: Vulcano-plots of all methylation regions analysed between CVID patients and healthy controls. *Our analyses included 805,722 methylation regions for CD3⁺ cells, and 776,336 methylation regions for CD14⁺ cells. A methylation region was considered a differentially methylated regions (DMR) only if the region showed a Benjamini–Hochberg adjusted p-value <0.05 and a minimum absolute methylation difference of 10%. Orange dashed lines show thresholds. Light purple dots show methylation regions below at least one threshold, with dark purple dots showing DMRs that were above both thresholds.*

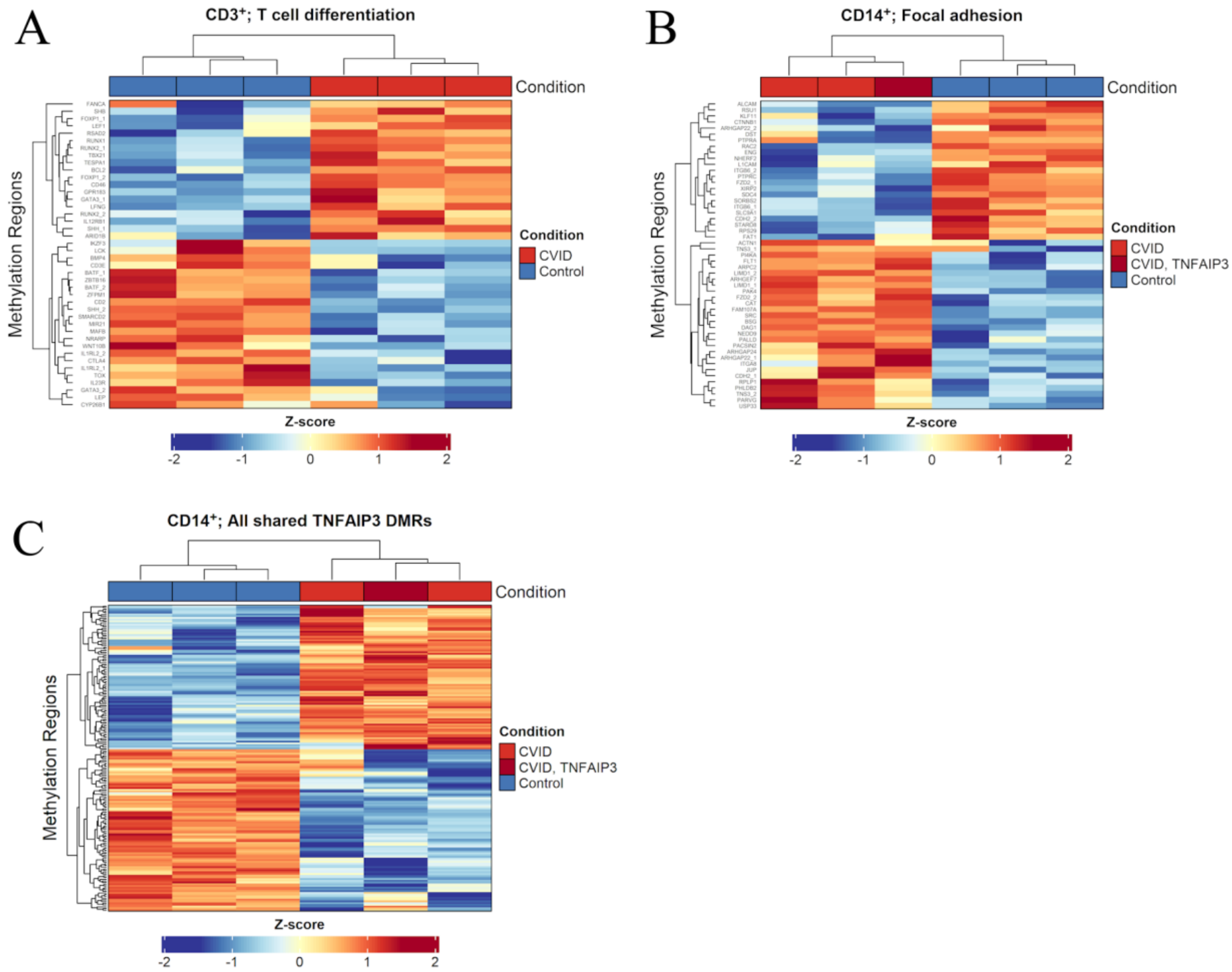

Supplementary Figure S8: Additional heatmaps from over-representation analysis. A) *Individual methylation levels in DMRs involved in the enriched T cell differentiation pathway in CD3*$^{+}$. ***B)*** *Individual methylation levels in DMRs involved in the focal adhesion pathway in CD14*$^{+}$. ***C)*** *Methylation regions in CD14*$^{+}$ *that could potentially drive group levels results in the over representation analysis between CVID and controls.*

Supplementary Tables

Supplementary Table S1: UniProt description of Wnt-related proteins (6).

Gene name	UniProt entry	Function
DKK1	O94907	DKKs play an important role in vertebrate development, where they locally inhibit Wnt regulated processes such as antero-posterior axial patterning, limb development, somitogenesis and eye formation. In the adult, Dkks are implicated in bone formation and bone disease, cancer and Alzheimer disease (PubMed:17143291). Inhibits the pro-apoptotic function of KREMEN1 in a Wnt-independent manner, and has anti-apoptotic activity (By similarity).
DKK3	Q9UBP4	Antagonizes canonical Wnt signaling by inhibiting LRP5/6 interaction with Wnt and by forming a ternary complex with the transmembrane protein KREMEN that promotes internalization of LRP5/6. DKKs play an important role in vertebrate development, where they locally inhibit Wnt regulated processes such as antero-posterior axial patterning, limb development, somitogenesis and eye formation.
FRZB	Q92765	Soluble frizzled-related proteins (sFRPS) function as modulators ofWnt signaling through direct interaction with Wnts. They have a role in regulating cell growth and differentiation in specific cell types. SFRP3/FRZB appears to be involved in limb skeletogenesis. Antagonist of Wnt8 signaling. Regulates chondrocyte maturation and long bone development.
RSPO3	Q9BXY4	Activator of the canonical Wnt signaling pathway by acting as aligand for LGR4-6 receptors, which acts as a key regulator of angiogenesis. Upon binding to LGR4-6 (LGR4, LGR5 or LGR6), LGR4-6 associate with phosphorylated LRP6 and frizzled receptors that are activated by extracellular Wnt receptors, triggering the canonical Wnt signaling pathway to increase expression of target genes. Also regulates the canonical Wnt/beta-catenin-dependent pathway and non-canonical Wnt signaling by acting as an inhibitor of ZNRF3, an important regulator of the Wnt signaling pathway. Acts as a ligand for frizzled FZD8 and LRP6. May negatively regulate the TGF-beta pathway (PubMed:21727895, PubMed:21909076, PubMed:22615920).
WIF1	Q9Y5W5	Binds to WNT proteins and inhibits their activities. May be involved in mesoderm segmentation.

References

1. Yang M, Kaarbø M, Myhre V, Reims HM, Karlsen TH, Wang J, et al. Altered genome-wide DNA methylation in the duodenum of common variable immunodeficiency patients. J Clin Immunol [Internet]. 2024;44. Available from: https://doi.org/10.1007/s10875-024-01726-5
2. Perez G, Barber GP, Benet-Pages A, Casper J, Clawson H, Diekhans M, et al. The UCSC genome browser database: 2025 update. Nucleic Acids Research [Internet]. 2024 Oct;53(D1):D1243–9. Available from: https://doi.org/10.1093/nar/gkae974
3. Park IAX Joon AND Munagala. Interferon signature in the blood in inflammatory common variable immune deficiency. PLOS ONE [Internet]. 2013 Sep;8(9):null. Available from: https://doi.org/10.1371/journal.pone.0074893
4. Rodríguez-Ubreva J, Calafell-Segura J, Calvillo CL, Keller B, Ciudad L, Handfield LF, et al. COVID-19 progression and convalescence in common variable immunodeficiency patients show dysregulated adaptive immune responses and persistent type i interferon and inflammasome activation. Nature communications. 2024;15(1):10344.
5. Rodríguez-Ubreva J, Arutyunyan A, Bonder MJ, Del Pino-Molina L, Clark SJ, Calle-Fabregat C de la, et al. Single-cell atlas of common variable immunodeficiency shows germinal center-associated epigenetic dysregulation in b-cell responses. Nature communications. 2022;13(1):1779.
6. Ahmad S, Jose da Costa Gonzales L, Bowler-Barnett EH, Rice DL, Kim M, Wijerathne S, et al. The UniProt website API: Facilitating programmatic access to protein knowledge. Nucleic Acids Research [Internet]. 2025 May;53(W1):W547–53. Available from: https://doi.org/10.1093/nar/gkaf394
